# Metagenomic Analysis Using Phylogenetic Placement—A Review of the First Decade

**DOI:** 10.3389/fbinf.2022.871393

**Published:** 2022-05-26

**Authors:** Lucas Czech, Alexandros Stamatakis, Micah Dunthorn, Pierre Barbera

**Affiliations:** ^1^ Department of Plant Biology, Carnegie Institution for Science, Stanford, CA, United States; ^2^ Computational Molecular Evolution Group, Heidelberg Institute for Theoretical Studies, Heidelberg, Germany; ^3^ Institute for Theoretical Informatics, Karlsruhe Institute of Technology, Karlsruhe, Germany; ^4^ Natural History Museum, University of Oslo, Oslo, Norway; ^5^ Independent Researcher, Bisingen, Germany

**Keywords:** phylogenetic placement, evolutionary placement, phylogenetics, metagenomics, metabarcoding, species diversity, taxonomic assignment, sequence identification

## Abstract

Phylogenetic placement refers to a family of tools and methods to analyze, visualize, and interpret the tsunami of metagenomic sequencing data generated by high-throughput sequencing. Compared to alternative (e. g., similarity-based) methods, it puts metabarcoding sequences into a phylogenetic context using a set of known reference sequences and taking evolutionary history into account. Thereby, one can increase the accuracy of metagenomic surveys and eliminate the requirement for having exact or close matches with existing sequence databases. Phylogenetic placement constitutes a valuable analysis tool *per se*, but also entails a plethora of downstream tools to interpret its results. A common use case is to analyze species communities obtained from metagenomic sequencing, for example via taxonomic assignment, diversity quantification, sample comparison, and identification of correlations with environmental variables. In this review, we provide an overview over the methods developed during the first 10 years. In particular, the goals of this review are 1) to motivate the usage of phylogenetic placement and illustrate some of its use cases, 2) to outline the full workflow, from raw sequences to publishable figures, including best practices, 3) to introduce the most common tools and methods and their capabilities, 4) to point out common placement pitfalls and misconceptions, 5) to showcase typical placement-based analyses, and how they can help to analyze, visualize, and interpret phylogenetic placement data.

## 1 Introduction

Advances in sequencing technologies enable the broad sequencing of genetic material in environmental samples (Edwards and Holt, 2013; Sunagawa et al., 2013), for instance, from water (Karsenti et al., 2011; Giner et al., 2016; Lacoursière-Roussel et al., 2016), soil (Dupont et al., 2016; Mahé et al., 2017), and air (Clare et al., 2022), which is known as environmental DNA (eDNA, Deiner et al., 2017; Ruppert et al., 2019), or from the human body (Curtis et al., 2012; Methé et al., 2012; Matsen, 2015; Wang et al., 2015) and other sources (Hanson et al., 2016; ElRakaiby et al., 2019; Gohli et al., 2019; Lorimer et al., 2019). Crucially, this enables the ecological survey of a community of organisms in their immediate environment (i. e., *in situ*), and allows to directly study the genetic composition of species communities (from viruses to megafauna); a field known as metagenomics (Thomas et al., 2012; Escobar-Zepeda et al., 2015; Oulas et al., 2015; Lindgreen et al., 2016).

Metagenomic data typically stem from so-called *High-Throughput Sequencing* (HTS, Pettersson et al., 2009; Reuter et al., 2015; Goodwin et al., 2016) technologies, such as *Next Generation Sequencing* (NGS, Logares et al., 2012; Mardis, 2013), as well as later generations (Niedringhaus et al., 2011; Pareek et al., 2011; Mignardi and Nilsson, 2014; Heather and Chain, 2016; Mardis, 2016). For a sample of biological material, these technologies typically produce thousands to millions or even billions of short genetic sequences (also called “reads”) with a length of some hundred base pairs length each. Over the past decades, decreasing costs and increasing throughput of sequencing technologies have caused an exponential growth in sequencing data (Muir et al., 2016), which has now passed the peta-scale barrier (Katz et al., 2022).

A major analysis step in metagenomic studies is to characterize the reads obtained from an environment by means of comparison to *reference sequences* of known species (Desai et al., 2012). A straight-forward way to accomplish this is to quantify the similarity between the reads and reference sequences. We obtain an indication of possible novelty if the sequence similarity to known species is low (Temperton et al., 2012; Peabody et al., 2015). However, such approaches do not provide the user with the evolutionary context of the read, and have been found to incorrectly identify sequences (Koski and Golding, 2001; Clemente et al., 2011; Mahé et al., 2017).

Instead, general phylogenetic methods can be used directly to classify and characterize the reads, providing highly accurate and information-rich results (Brady and Salzberg, 2009; Segata et al., 2012; Truong et al., 2015; Jamy et al., 2019; Beghini et al., 2021). However, trying to resolve the phylogenetic relationships between millions of short reads and the given reference sequences represents a significant computational challenge. Furthermore, as most phylogenetic methods require an *alignment* of sequences, metagenomic data can often not be used directly, as whole-genome reference data might not be available or computationally intractable. Instead, specific *marker genes* can be targeted (or filtered from the metagenomic data), which are genetic regions that are well-suited for differentiating between species (Ren et al., 2016). The use of marker genes to identify species is called *DNA (meta-)barcoding* (Deiner et al., 2017; Hebert et al., 2003; Savolainen et al., 2005; Kress and Erickson, 2008); see Section 2.2 for details.

A powerful and increasingly popular class of methods to identify and analyze diverse (meta-)genomic (barcode) data is the so-called *phylogenetic placement* (or *evolutionary placement*) of genetic sequences onto a given fixed phylogenetic *reference tree*. By placing unknown, anonymous sequences (in this context called *query sequences*) into the evolutionary context of a tree, these methods allow for the taxonomic assignment of the sequences (i. e., the association of genomic reads to existing species, for example Auladell et al., 2019; Jamy et al., 2019; Hleap et al., 2021). Moreover, they can also provide information on the evolutionary relationships between these query sequences and the reference species/sequences, and thus go beyond simple species identification. Phylogenetic placement has found applications in a variety of situations, such as data cleaning and retention (Mahé et al., 2017), inference of new clades (Dunthorn et al., 2014; Bass et al., 2018), estimation of ecological profiles (Keck et al., 2018), identification of low-coverage genomes of viral strains (Mühlemann et al., 2020), phylogenetic analysis of viruses such as SARS-CoV-2 (Morel et al., 2020; Turakhia et al., 2021), and in clinical studies of microbial diseases (Srinivasan et al., 2012).

When analyzing the resulting data, there are two complementary interpretations of phylogenetic placement: 1) as a set of individual sequences, placed with respect to the reference phylogeny, e. g., for taxonomic assignment, phylo-geographic tracing, or even possible clinical relevance; 2) as a combined distribution of sequences on the tree, characterizing the sampled environment at a given point in time or space to examine the composition of a species community as a whole, for instance as a means of sample ordination and visualization, and association with environmental variables.

In this review, we provide an overview of existing methods to conduct phylogenetic placement, as well as post-analysis methods for visualization and knowledge inference from placement data. We also discuss some practical aspects, such as common pitfalls and misconceptions, as well as caveats and limitations of these methods. We mainly refer to metagenomic input data (or more accurately, metabarcoding data, see below for details) as it represents the most common use case, but also highlight some alternative use cases where phylogenetic placement is employed for other types of sequence data.

## 2 Phylogenetic Placement

### 2.1 Overview and Terminology

The modern approach to phylogenetic tree inference is based on molecular sequence data, and uses stochastic models of sequence evolution (Arenas, 2015) to infer the tree topology and its branch lengths (Felsenstein, 2004; Yang, 2006). Note that the computational cost to infer the optimal tree under the given optimality criterion grows super-exponentially in the number of sequences (Felsenstein, 2004). In addition, large trees comprising more than a couple of hundred sequences are often cumbersome to visualize, rendering the approach challenging for current (e. g., metagenomic) large datasets. Furthermore, the lack of phylogenetic signal contained in the short reads of most HTS technology usually does not suffice for a robust tree inference (Dunthorn et al., 2014; Bininda-Emonds et al., 2001; Moret et al., 2002; von Mering et al., 2007). Hence, *phylogenetic placement* emerged from the demand to obtain phylogenetic information about sequence sets that are too large in number and too short in length to infer comprehensive phylogenetic trees (Matsen et al., 2010; Berger et al., 2011). In a metagenomic context, a set of sequences obtained from an environment such as water, soil, or the human body, is here called a *sample*. This is often the data that we intend to place, and might have further metadata associated with it, e. g., environmental factors/variables such as temperature or geo-locations where the sample was taken.

Generally, the input of a phylogenetic placement analysis is a phylogenetic *Reference Tree* (RT) consisting of sequences spanning the genetic diversity that is expected in the sequences to be placed into the tree. The tree can be rooted or unrooted; in the latter case however, a “virtual” root (or top-level trifurcation) is used in the computation as a fixed point of reference (Czech et al., 2019). Then, for a single sequence (e. g., a short read), in this context called a *Query Sequence* (QS), the goal of phylogenetic placement is to determine the branches of the RT to which the QS is most closely evolutionarily related. Note that the RT is kept fixed, that is, the QSs are not inserted as new branches into the tree, but rather “mapped” onto its branches. Hence, the phylogenetic relationships *between* individual QSs are not resolved.

**FIGURE 1:**
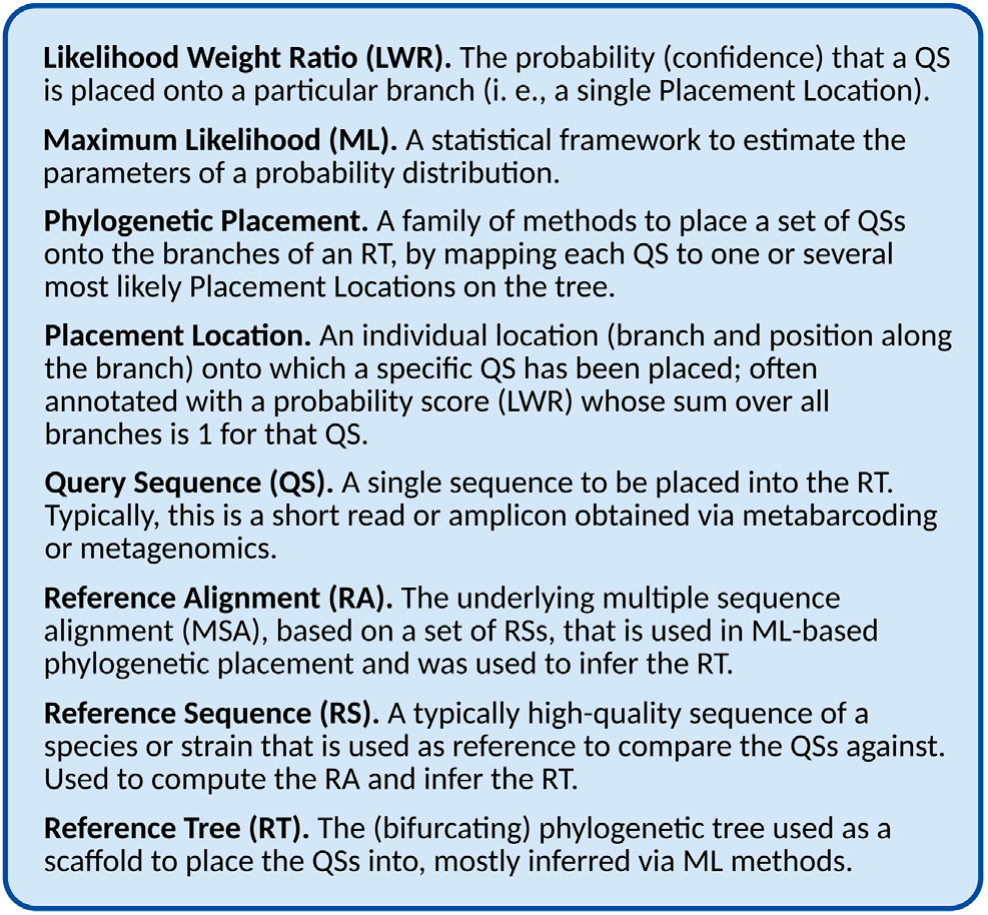
Glossary and abbreviations.

This is the key insight that makes it possible to efficiently compute the placement of large numbers of QSs. By only determining the evolutionary relationship between the sequences of the RT and each individual QS, the process can be efficiently parallelized, and the required processing time scales linearly in the number of QS. Furthermore, this allows us to consider multiple branches as potential *Placement Locations* for a given QS, representing uncertainty in the placement, often expressed as a probability (or confidence) of the QS being placed on that branch. This uncertainty might result from weak phylogenetic signal, or might indicate some other issue with the data, as explained later. In Maximum-Likelihood (ML) based placement (see Section “Maximum Likelihood Placement” for details), these probabilities are computed as the *Likelihood Weight Ratio* (LWR) resulting from the evaluation of placing the QS attached to an additional (hypothetical) branch into the tree. Hence, for historic reasons, the probability of a placement location (one QS placed on a specific branch) is often called its LWR, and for a given QS, the sum of LWRs over all branches is 1 (equivalent to the total probability). See Figure 1 for a glossary of the terminology, and see Table 1 for an overview of different placement tools, and which of the aforementioned quantities they can compute.

**TABLE 1:** General purpose placement tools. This table compares the features of the general purpose (i. e., not use-case specific) phylogenetic placement tools. Columns are as follows. Alignment: Does the tool need the QSs to be aligned against the reference alignment? Multiple: Does the tool produce multiple placement locations per QS, or just a single (best) one? Uncertainty: Is there some measure of uncertainty (such as LWR) assigned to each placement location? Branch Length: Does the tool compute the involved branch lengths at each placement location for each QS.

Placement Tool	Alignment	Multiple	Uncertainty	Branch Lengths
pplacer	yes	yes	yes	yes
RAxML-EPA	yes	yes	yes	yes
EPA-ng	yes	yes	yes	yes
RAPPAS	no	yes	yes	no
APPLES	no	no	no	yes
App-SpaM	no	no	no	yes

**FIGURE 2:**
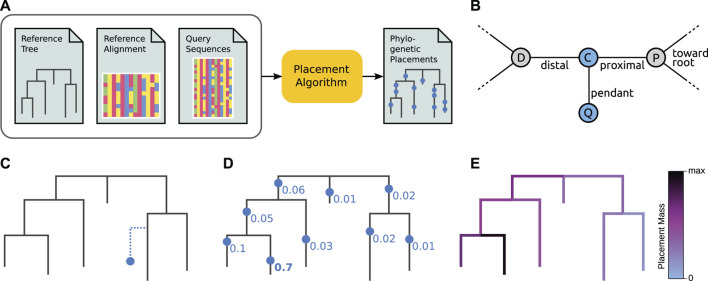
Overview of phylogenetic placement. Here, we show the typical process, focused on ML-based placement. For the sake of simplicity, we here omit heuristics and other algorithmic improvements. Alignment-free placement works conceptually in an analogous way, but does not compute tree likelihoods. **(A)** Pipeline and data flow. The input to phylogenetic placement are the Reference Tree (RT) and its corresponding Reference Alignment (RA), as well as the set of Query Sequences (QSs) that we are interested in. The placement algorithm computes potential placement locations of a QS on the branches of the RT, for each QS in the input. **(B)** Terminology. The nodes D and P belong to the Reference Tree (RT). When placing a Query Sequence (QS), the branch between these nodes is split into two parts by a temporary new node C, which serves as the attachment point for another temporary new node Q that represents the QS. Note that these two new nodes are only conceptually inserted into the RT–they represent the mapping of the QS onto that branch. The *pendant* branch leads to Q. The original branch is split into the *proximal* branch, which leads towards the (possibly virtual) root of the RT, and the *distal* branch, which leads away from the root. **(C)** A single QS is placed onto a single branch (that is, one placement location). Vertical distances symbolize branch lengths. Note that the QS is located at a certain position along its Reference Tree branch (splitting that branch into distal and proximal parts), and has a (pendant) branch length of its own. At this step, ML-based placement computes the likelihood of the RT with the QS as a (temporary) extra branch. For one single QS, this step is then repeated at every branch of the tree. **(D)** Once the likelihoods of placing the QS onto every branch have been computed, the Likelihood Weight Ratios (LWRs) for this QS are computed. They express the confidence of placing the QS onto each branch, and can be interpreted as a probability distribution of the QS across the tree (and hence sum to one across all branches). In the image, we omit pendant branch lengths for the sake of simplicity. **(E)** The process is repeated for every QS, yielding an LWR-weighted “mapping” of each QS to each branch. We can visualize this as a cumulative distribution across all QSs on the tree, coloring branches according to the total sum of the LWRs at that branch over all QS. See Figure 4A for a real-world example of this.

In other words, phylogenetic placement can be thought of as an all-to-all mapping from QSs to branches of the RT, with a probability for each placement location, as shown in Figures 2D,E. We can however also interpret each such placement location *as if* it was an extra branch inserted into the RT, as shown in Figures 2B,C. In particular, maximum likelihood placement makes use of its underlying evolutionary model to also estimate the involved branch lengths that are altered through the insertion of a QS, see Figure 2B for details. This interpretation highlights the aspect of each individual QS being part of the underlying phylogeny. For example, this allows its taxonomic assignment to that clade of the reference tree where the QS shows the highest accumulated placement probability, as explained later.

#### 2.1.1 Misconceptions

In the existing literature, and from our experience in teaching the topic as well as supporting the users of our software, some concepts of phylogenetic placement are not always well explained or understood. Although we have introduced these concepts above already, we briefly address two common misconceptions here, for clarity.

Firstly, a common misconception is that the tree is amended by the QSs, that is, that new branches are added to the RT, and that the phylogenetic relationships of the QSs with each other are hence resolved. This is not the case; instead, the RT is kept fixed, the QSs are only aligned against the reference alignment, but not against each other (in ML placement), and the QSs are mapped only to the existing branches in the RT. This mapping *can* however be interpreted “as if” the QS was a new terminal node (leaf or tip) of the tree, usually inserted (or “grafted”) into the branch with the most probable placement location, which can be useful in some applications.

Secondly, a further common misconception is that a QS is only placed onto a single branch, or that only the best (most likely) placement location is taken as the result for each placed QS. Instead, each branch is seen as a potential placement location with a certain probability, which sum to one over the tree. It can however be useful to reduce the placement distribution of a QS to only its most probable placement location. Also, for practical reasons, typically not all locations are stored in the resulting file (or even considered in the computation by application of heuristics), as low probability locations can often be discarded to save storage space and downstream processing time; see Section “File Format” for details. Lastly, some placement methods do only output a single best placement, see Table 1.

In summary, phylogenetic placement yields a distribution of potential locations of where a QS could be attached in the RT–but it does not extend the RT by the QS with an actual branch.

#### 2.1.2 File Format

**FIGURE 3:**
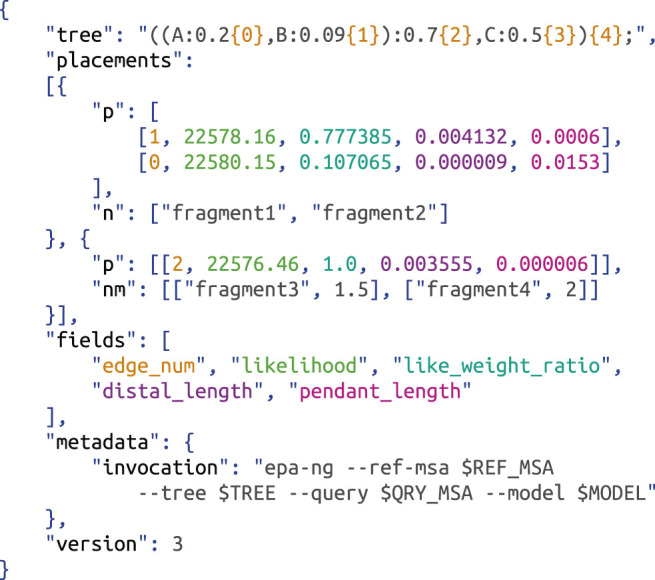
Jplace format for phylogenetic placement. The exemplary file consists of a reference "tree" in a custom Newick format that annotates edge numbers in curly brackets, followed by two pqueries, which is the term for combined lists of sequence names and their placement locations. The first pquery contains two placement locations ("p") for two query sequences ("n"), and the second contains a single location ("p") for two other sequences including their multiplicities/abundances ("nm"). The order to interpret the values per location is given via the "fields" list, and highlighted by colors here; additional "metadata" and a "version" of the file format can be given. Example adapted from (Matsen et al., 2012).

Placement data is usually stored in the so-called jplace format (Matsen et al., 2012), which is based on the json format (Bray, 2018; Douglas, 2018). See Figure 3 for an example. It uses a custom augmentation of the Newick format (Archie et al., 1986) to store the reference tree, where each branch is additionally annotated by a unique edge number, so that placement locations can easily refer to the branches. For each QS (named via the list "n"), the format then stores a set of possible placement locations (in the list "p"), where each location is described by the values: 1) "edge_num", which identifies the branch of this placement location, 2) "likelihood", which is used by maximum likelihood based placement methods, 3) "like_weight_ratio" (LWR), which denotes the probability (or confidence) of this placement location for the given QS, 4) "distal_length" and 5) "pendant_length", which are the branch lengths involved in the placement of the QS for the given placement location; see Figure 2B for an explanation of these lengths.

These five data fields are the standard fields of the jplace format; further fields can be added as needed. As noted above, typically not all placement locations for a given QS are stored in the file, as low probability placements unnecessarily increase the file size without providing substantial information; in that case, the sum of the stored LWR values might actually be smaller than 1.

The format furthermore allows for multiple names in the "n" list, as well as assigning a “multiplicity” to each such name (by using a list called "nm" instead of "n"). For instance, this allows to only store the placement locations for identical reads once, while keeping track of the original raw abundances of these reads or OTUs. A pair of a "n"/"nm" list and a "p" list is called a “pquery”, and describes a set of placement locations for one or more (identical) QSs. This structure is then repeated for each QS that has been placed.

To our knowledge, the genesis library (Czech et al., 2020) is the only general purpose toolkit for working with, and manipulating, placement data in jplace format. It also incorporates many of the downstream visualization and analysis techniques we describe later on. Some other tools that offer basic capability to work with jplace files are BoSSA (Lefeuvre, 2018), ggtree (Yu et al., 2017), and treeio (Wang et al., 2020), all of which can read jplace files for processing in R.

With the release of several placement tools that do not use the ML framework, see Section “Distance-Based Placement”, the jplace file format (Matsen et al., 2012) may require an update. The standard is written currently (as of version 3) with placement properties such as branch lengths and likelihood scores in mind, which do not translate well to other types of placement algorithms (pers. comm. with S. Mirarab, July 2020). Furthermore, it might be helpful to support sample names, multiple samples per file, and additional per-sample or even per-query annotations and other metadata in the file format. Being based on json, this can already be achieved now by adding these entries ad-hoc, but would lack support by parsers if not properly standardized.

### 2.2 Types of Query Sequences

In principle, any type of genetic sequence data can be subjected to placement, as long as the reference sequences span the genomic regions where the query sequences originate from. Apart from the availability of suitable reference sequences used to construct a reference tree (see Section “Sequence Selection”), the primary limiting factor is the extent to which a given placement tool supports the data. Currently, the majority of placement tools supports nucleotide (DNA/RNA) and amino acid (protein) data. Many placement methods require query reads to be aligned to the reference, i. e. they need to be homologs.

#### 2.2.1 Metabarcoding and Amplicons

For the above reasons, a common approach to obtain sequences is *metabarcoding* (Deiner et al., 2017; Hebert et al., 2003; Savolainen et al., 2005; Kress and Erickson, 2008). In metabarcoding, one or several *marker* or *barcoding* genes, such as 16S (Weisburg et al., 1991), 18S (Meyer et al., 2010), ITS, COI, etc. (Woese and Fox, 1977; Woese et al., 1990; Ji et al., 2013; Sunagawa et al., 2013) are typically chosen to compute the reference alignment, and appropriate primers are selected to enable metabarcode sequencing of the sample (Deiner et al., 2017). A marker gene should be universally present in the studied organisms, and ideally should only occur once in the genome of each organism (Dunthorn et al., 2014; Nguyen et al., 2014), i. e., be single-copy. In practice, marker genes often occur multiple times per genome, possibly requiring the need for copy number correction. A marker gene should exhibit sufficient between-species variation to distinguish them from each other, but show low within-species variation (Kress and Erickson, 2008). Using a metabarcoding approach has several advantages: it targets loci of interest and focuses the sequencing effort there (incidentally also limiting the size of the reference MSA), barcoding genes are typically well suited for phylogenetics (stable regions to aid alignment paired with variable regions to discriminate organisms), and the approach is generally cost-effective. Such approaches use amplicon sequencing (Peabody et al., 2015; Hugerth and Andersson, 2017), wherein only DNA originating from the targeted region is amplified using the Polymerase Chain Reaction (PCR, Bartlett and Stirling, 2003), thus yielding the subsequent sequencing of any remaining DNA fragments from other regions highly improbable. The resulting amplicon sequences have been shown to be well-suited for phylogenetic placement (Mahé et al., 2017; Janssen et al., 2018).

However, PCR-based amplifications are known to introduce biases in the abundance of the sequencing reads, as some fragments may be copied with a higher likelihood than others (Morgan et al., 2010; Logares et al., 2014). Similarly, a further bias that skews abundance results exists as different organisms may have a different number of copies of the targeted gene, ranging from single copies to 15 copies, depending on the organism (Lee et al., 2009). Some methods exist that attempt to account for copy number bias (Kembel et al., 2012; Angly et al., 2014; Pereira-Flores et al., 2019) as well as for PCR amplification bias (Love et al., 2016; Silverman et al., 2021).

When an untargeted sequencing approach is chosen instead (such as shotgun metagenomic sequencing), using a broader scope for the reference sequences may be advisable, such as using whole genome data. This might only be feasible for small genomes such as some viruses or mitochondrial DNA. Alternatively, a sensible approach is to filter out any reads that did likely not originate from the genetic regions that constitute the reference alignment. This can be achieved, for example, using hmmsearch from the HMMER-package (Eddy, 1995; Eddy, 1998), which allows the user to obtain a list of reads that have an alignment score above a given threshold. Similarly, so-called _mi_tags (Logares et al., 2014) represent a shotgun-based alternative to amplicon sequencing.

Recently, placement methods have emerged that do not require the alignment of query sequences to a reference, and some do not even require the references to be aligned against each other (see Section “Distance-Based Placement”). However, establishing that query reads and reference sequences are homologous is still necessary.

#### 2.2.2 Sequencing Technologies

A further consideration is the choice of sequencing technology, with the primary property being the length of the resulting sequencing reads. So far, the vast majority of studies utilizing phylogenetic placement have relied on short-read sequencing technologies such as NGS, using by now well established protocols to perform broad low-cost sequencing (van Dijk et al., 2014). However, this approach produces very short (150-400 nucleotide) reads, that typically only cover fragments of a reference gene. For universal single-copy markers, this can limit their applicability to phylogenetics due to the lower information content. However, the approach has been applied successfully to other types of data (Piredda et al., 2021; Cardoni et al., 2022).

More recent sequencing technologies, called third generation sequencing, or long-read sequencing (LRS), yield individual reads that cover entire genes, or even entire genomes (Amarasinghe et al., 2020). While placement was originally developed for short read sequencing, longer read lengths typically increase the phylogenetic signal contained in reads, thus increasing the reliability of phylogenetic methods. Indeed, such sequence data have been shown to overcome this fundamental hurdle to phylogenetically resolving the relationships between query sequences that originally gave rise to phylogenetic placement (Jamy et al., 2019).

An emerging third way to obtain longer reads is to combine short reads into longer so-called Synthetic Long-Reads (SLRs), which have been used successfully to characterize metagenomes (Sharon et al., 2015; Kuleshov et al., 2016) and which improve upon short-read metabarcoding approaches for taxonomic classification (Jamy et al., 2019; Ritter et al., 2020; Jeong et al., 2021).

Related to this is the assembly of genomes from metagenomic sequences (MAGs, Tyson et al., 2020), a technique which has recently been shown to reliably obtain multi-loci data from highly diverse data sources and environments (Parks et al., 2017). MAGs may be a beneficial input for phylogenetic placement, especially for methods that are able to directly handle such assemblies in their entirety (Metin et al., 2021). Other placement methods may also benefit from sequence assemblies when combined with marker gene extraction, as it potentially increases the number of viable query sequences.

#### 2.2.3 Clustering

Once the wet-lab sequencing strategy has been determined, a user eventually obtains a (typically large) set of sequences. After quality control, a potential next step is to consider if, and how, to cluster these raw sequences in order to reduce the amount of data that has to be processed, often at the cost of losing information. Common choices include clustering by similarity threshold (≥97%) resulting in Operational Taxonomic Units (OTUs, Blaxter et al., 2005; Edgar, 2010; Fu et al., 2012; Westcott and Schloss, 2015; Rognes et al., 2016), more strictly based on single nucleotide differences resulting in Amplicon Sequencing Variants (ASVs, Callahan et al., 2016), or more recent alternatives such as SWARM clustering (Mahé et al., 2021). These methods are most commonly used for clustering reads from marker regions, and hence applicable in the placement context; for a comprehensive review of clustering methods, see (Zou et al., 2020).

If possible, it is recommended to avoid clustering, in order to retain potential phylogenetic signal; this choice however also depends on study design and goals. However, even if sequences are not clustered, we strongly recommend dereplication, that is, removal of exact (strict) duplicates of sequences, to avoid unnecessary redundant computations. For the same reason, sequence dereplication is also useful when pooling the sequences from multiple samples together and placing the resulting set via a single placement run. Tools that offer this capability include USEARCH (Edgar, 2010), and VSEARCH (Rognes et al., 2016), as well as the placement-specific chunkify command in gappa (Czech et al., 2020).

#### 2.2.4 Outgroup Rooting

Finally, an often overlooked source of query sequences are high-quality reference sequence databases. Here, the use-case of placement shifts away from taxonomic assignment: instead such data can be used to attempt an outgroup rooting of an existing tree, using already classified sequences (Hubert et al., 2014; Liede-Schumann et al., 2020; Morel et al., 2020). The result of placement, in this case, is a set of suggested branches on which to root the tree, including a probability estimate for each root placement onto each branch (Liede-Schumann et al., 2020).

### 2.3 Reference Sequences, Alignment, and Tree

The phylogenetic reference tree (RT), inferred from a set of reference sequences (RSs) using their alignment (*Reference Alignment*, RA), is the foundation and scaffold for conducting phylogenetic placement. Ideally, to avoid duplicating work, to ensure high quality, and to provide stable points of reference for comparison between studies, suitable reference trees should be provided by the respective research/organismal communities. First efforts for microbial eukaryotes are on their way (Berney et al., 2017; Del Campo et al., 2018; Rajter and Dunthorn, 2021; Rajter et al., 2021), although some of these are not designed explicitly for phylogenetic placements, but more taxonomic groups will follow. Recently, efforts have also been made to produce reference trees for higher order animals, such as fish (Collins et al., 2021). As references are however not yet available for all taxonomic groups, we here provide an overview of the process (see also Mahé et al., 2017, Rajter et al., 2021, for practical examples).

#### 2.3.1 Sequence Selection

As phylogenetic placement cannot infer evolutionary relationships below the taxonomic level of the reference tree, the first step is the selection of suitable RSs, which should 1) cover the diversity that is expected in the query sequences (QSs), and 2) be well-established and representative for their respective clades to facilitate meaningful interpretation. In order to capture unexpected diversity and potential outliers, it can be advantageous to include a wider range of sequences as well (Mahé et al., 2017), or to run preliminary tests and filtering (placement- or similarity-based) with a broad reference to ensure that all diversity in the QSs is accounted for.

In many cases, the selection process is (unfortunately) labor-intense, as it requires hand-selecting known sequences from reference databases such as SILVA (Pruesse et al., 2007; Quast et al., 2013; Yilmaz et al., 2014), NCBI (Benson et al., 2009; Sayers et al., 2009), GREENGENES (DeSantis et al., 2006; McDonald et al., 2012), or RDP (Wang et al., 2007; Cole et al., 2014). This manual process however also often provides the highest quality, and allows to optimally assemble the RSs for a given project. See also (Balvočiūtė and Huson, 2017) for a comparison of these databases.

Important selection criteria are the number of sequences to be selected, as well as their diversity; both of which depend on the study design and goals. Generally, a number of RSs in the order of hundreds to a few thousands has shown to provide enough coverage for most QS datasets, while still being small enough to properly visualize their phylogeny and to conduct all necessary computations in reasonable time. Often, it is sufficient to include a single species to represent a whole clade (Rajter and Dunthorn, 2021). Depending on the types of downstream analyses, it can be a disadvantage to select sequences that are too similar to each other (i. e., closely related species, or different strains of the same species), as this can spread the placement distribution across nearby branches. In other words, placements with similar probability in many branches are mostly a consequence of reference alignment regions for which large subtrees contain (almost) identical sequences. This is however expected when conducting taxonomic assignment at species or below-species level, and the reference should be built with the targeted taxonomic resolution in mind.

On the other hand, if the QSs contain enough phylogenetic signal (e. g., when using long reads, whole genome data, or when the target gene has sufficient variability), including multiple representatives of a taxonomic group might allow to obtain more finely resolved placements. For example, in short genomes such as HIV or arthropod mitochondria, where mutations are not concentrated in specific regions but spread all over the genome, reads matching a reference alignment region likely show a decent amount of variation, making placements exploitable (Linard et al., 2020).

Lastly, the RSs need to at least span the genomic region that the QSs come from. For a more robust inference of the RT however, it can be advantageous to include a larger region with more phylogenetic signal. Theoretically, if one wanted to place shotgun sequences from entire genomes, whole-genome RSs would be needed.

As an alternative to manual selection, the Phylogenetic Automatic Reference Tree (PhAT, Czech et al., 2018) is a method that uses reference taxonomic databases to select suitable RSs which represent the diversity of (subsets of) the database. In cases where taxonomic resolution at the species-level does not require expert curation, the PhAT method can provide a basis for rapid data exploration, and help to obtain an overview of the data and its intrinsic diversity.

#### 2.3.2 Reference Alignment Computation

Next, for ML-based tree inference and placement, the RSs need to be aligned against each other to obtain the reference alignment (RA). Typically, this is conducted with *de novo* multiple sequence alignment tools such as T-Coffee (Notredame et al., 2000), MUSCLE (Edgar, 2004), MAFFT (Katoh et al., 2002), and others; see (Kemena and Notredame, 2009; Pervez et al., 2014; Chatzou et al., 2016) for reviews. Recently, MUSCLE v5 introduced an interesting new approach that generates alignment ensembles to capture alignment uncertainty (Edgar, 2021, preprint). In the ML framework, the QSs also need to be aligned against the RA, see next section.

#### 2.3.3 Tree Inference

Finally, given the RA, a phylogenetic tree of the RSs is inferred, which is henceforth used as the reference tree (RT); see (Kapli et al., 2020) for a general review on this topic. In theory, any method that yields a fully resolved (bifurcating) tree is applicable, e. g., neighbor joining (Saitou and Nei, 1987), maximum parsimony (Sankoff, 1975), or Bayesian inference (Holder and Lewis, 2003; Yang, 2006). In practice however, maximum likelihood (ML) tree inference (Yang, 2006; Dhar and Minin, 2016) is preferred, in particular when using ML-based placement, as otherwise inconsistencies in the assumed models of sequence evolution can affect placement accuracy. To this end, common software tools include IQ-TREE (Nguyen et al., 2015), FastTree2 (Price et al., 2010), and RAxML (Stamatakis, 2014; Kozlov et al., 2019); see (Zhou et al., 2018) for a review and evaluation of ML-based tree inference tools. An open research question in this context is how to incorporate uncertainty in the tree inference (and in the alignment computation) with phylogenetic placement (Huelsenbeck et al., 2001; Ronquist, 2004; Edgar, 2021).

#### 2.3.4 Alignment of Query Sequences

For many placement methods, the query sequences need to be aligned against the reference alignment. In principle, *de novo* alignment methods can be deployed to obtain a comprehensive alignment of both the reference and query sequences. These tools are however not intended for HTS data, and are not well suited for handling the heterogeneity of phylogenetic placement data, with (typically) longer, curated, high-quality reference sequences, and short lower-quality reads (query sequences).

Hence, with the rise of high-throughput sequencing, specialized tools have been developed that extend a given (reference) alignment without fully recomputing the entire alignment. In the context of phylogenetic placement, there are two additional advantages that can be exploited to improve efficiency: 1) query sequences only need to be aligned against the reference, but not against each other (as their phylogenetic relationship is not resolved during placement), and 2) insertions into the reference that result from aligning a QS against the reference can be omitted as they do not contain any phylogenetic signal for the placement of the QS.

In the simplest case, only the reference alignment and query sequences are required as input. For instance, the hmmalign command of HMMER (Eddy, 1995; Eddy, 1998) can align query sequences to the reference alignment using a profile Hidden Markov Model (HMM) built from the reference alignment. Note that the option -m has to be set in order to not insert columns of gaps into the reference. Alternatively, the mafft command ‐‐addfragments (Katoh and Frith, 2012) uses an internally constructed guide tree built from a pairwise distance matrix of the reference alignment to aid the alignment process; here, the option ‐‐keeplength has to be set to not add columns of gaps to the reference.

Furthermore, the PAPARA tool (Berger and Stamatakis, 2011; Berger and Stamatakis, 2012) can be used that was specifically developed to target phylogenetic placement. It takes the RT as additional input, and uses inferred ancestral sequences at the inner nodes of the tree to improve the alignment process. Here, the option -r has to be set to not insert columns of gaps into the reference. Similarly, PAGAN (Löytynoja et al., 2012) also utilizes the information in the reference tree, but it *does* extend the reference alignment with gaps as needed for the query sequence, causing higher computational effort during placement.

Note that typically, read mapping tools such as BOWTIE2 (Langmead and Salzberg, 2012) or BWA (Li and Durbin, 2009; Li and Durbin, 2010) are not recommended for phylogenetic placement, as they expect low-divergent sequences as input, e. g., from a single species.

### 2.4 General Purpose Placement Methods

Once initial tasks such as reference tree creation and sequence alignment are completed, the actual placement can commence. There exist several distinct algorithmic approaches for conducting the core part of phylogenetic placement, which we introduce here; see Table 1 for an overview.

#### 2.4.1 Maximum Likelihood Placement

Maximum Likelihood (ML) is a statistically interpretable and robust general inference framework, and one of the most common approaches for phylogenetic tree inference (Felsenstein, 2004; Yang, 2006; Dhar and Minin, 2016). It works by searching through the super-exponentially large space of potential tree topologies for a given set of sequences (taxa), and computing the phylogenetic likelihood of the sequence data of these taxa being the result of the evolutionary relationships between the taxa as described by each potential tree, while also computing branch lengths of the tree. The result of this inference is the tree topology one is able to find using some heuristic search strategy that best (most likely) “explains” the underlying sequence data. Due to the NP-hardness of the tree search problem, the best tree one can find might not be the globally best one.

To calculate this likelihood, ML methods use statistical models of sequence evolution that describe substitutions between sequences (insertions and deletions are mostly ignored; it is hence also called a substitution model), see (Arenas, 2015) for a review. Consequently, the estimated parameters of these models are an inherent property of the resulting phylogenetic tree. The choice of model parameters also directly informs the specific branch lengths of a tree, interpreting a tree under a different set of model parameters thus may lead to inconsistencies. Therefore, under the ML framework, we strongly recommend to use the same substitution model and parameters for tree inference and for phylogenetic placement.

Based on the general ML tree inference framework, ML-based phylogenetic placement works in two steps: First, the QSs are aligned against the RA as described above, and second, using the resulting comprehensive alignment with both reference and query sequences, the QSs are placed on the RT using the maximum likelihood method to evaluate possible placement locations (Matsen et al., 2010; Stark et al., 2010; Berger et al., 2011).

Standard methods used in ML tree inference use search heuristics to explore some possible tree topologies for a given set of sequences. Instead, for a given QS, ML-based placement only searches through the branches of the reference tree (RT) as potential placement locations for the QS. That is, each branch of the RT is evaluated as a placement location, and branch lengths of the involved branches are optimized, following the same approaches as for *de novo* tree inference. However, the distal and proximal branch lengths of the placement (see Figure 2B for details) are typically re-scaled, so that their sum is equal to the original branch length in the RT. Finally, the phylogenetic likelihood of the tree with the QS amended as a temporary extra taxon is calculated.

For each QS and each branch of the RT, this process yields a likelihood score (which is stored in the jplace format, see Section “File Format”). The Likelihood Weight Ratio (LWR) of a placement location is then computed as the ratio between this likelihood score and the sum over all likelihood scores for the QS across the entire tree (von Mering et al., 2007; Strimmer and Rambaut, 2002). These likelihood scores sum to one across all branches, and hence express the confidence (or probability) of the QS being placed on a given branch.

The first two tools to conduct phylogenetic placement in an ML framework were the simultaneously published (as preprints) pplacer (Matsen et al., 2010) and RAxML-EPA (Berger et al., 2011). Both build on the same general ML concepts, but use different strategies for improving computational efficiency, e. g., by heuristically limiting the number of evaluated branches (potential placement locations). Additionally, pplacer offers a Bayesian placement mode. The more recent EPA-ng (Barbera et al., 2018) tool combines features from both pplacer and RAxML-EPA, is substantially faster and more scalable on large numbers of cores, and hence is the recommended tool for ML-based placement.

#### 2.4.2 Ancestral-Reconstruction-Based Placement

Recently, multiple methods were introduced that do not rely on aligning query sequences to a reference MSA. The first such group of methods is based on reconstructing ancestral states at interior nodes of the reference tree, again using an ML framework. From these ancestral sequences, *k*-mers are generated and associated with the branches of the reference tree. Subsequently, phylogenetic placement is performed by comparing the constituent *k*-mers of a QS with the set of *k*-mers indexing the reference tree branches, thereby obviating the need for QS alignment. This is the general approach used in both RAPPAS (Linard et al., 2019) and LSHplace (Brown and Truszkowski, 2012).

It should be noted that using this procedure, distal and pendant branch lengths of a given RT branch are determined during the association of *k*-mers with RT branches, meaning that all placements on a given branch have the same fixed location. This means that an additional step to conduct branch length optimization that is not directly offered by RAPPAS or LSHplace may be required to obtain more realistic placement branch lengths. RAPPAS however does produce multiple placements per QS and calculates a confidence measure akin to the LWR, yielding a distribution for placing a single QS onto different branches of the tree.

#### 2.4.3 Distance-Based Placement

Finally, the most recent placement approaches utilize methods from distance-based phylogenetic inference.

For example, APPLES (Metin et al., 2019) is based on the least-squares criterion for tree reconstruction (Felsenstein, 2004). For a given tree, the least-squares method calculates the difference between the pairwise sequence distances and the pairwise patristic distances (i. e., the path lengths between two leaves). A least-squares optimal tree is the tree for which this difference is minimized. In APPLES, this criterion is used to score possible placement locations of a QS on an existing tree, returning the branch which minimizes the between-distances difference. A key advantage of the least-squares approach is its ability to efficiently handle reference trees with hundreds of thousands of leaves, which is currently not computationally feasible using ML methods. Further, the method does not require an alignment of the sequences involved, requiring only a measure of pairwise distance between them. Note however that as these methods still require a reference tree, computing a reference MSA may still be needed, unless the tree is inferred via distance-based methods as well. Consequently, even unassembled sequences, such as genome skims (Dodsworth, 2015), may be used both as reference and query sequences. Recently, an updated APPLES-2 was published that further improves upon the scalability and accuracy of the tool (Metin et al., 2021). Note also that APPLES can take as input, but does not require, aligned sequences.

The most recent alignment-free method is App-SpaM (Blanke and Morgenstern, 2021). It utilizes the concept of a spaced-word, which can be understood as a type of *k*-mer for which only some characters have to be identical for two subsequences to be considered as having the same *k*-mer. This relaxed equality definition is informed by a binary pattern, indicating for each site of a spaced word whether it should be taken into account (1) or disregarded (0). Building on this, the tool calculates pairwise distances between a QS and the RSs based on the number of shared spaced-words. Subsequently, the tool identifies the placement branch of a QS as either the terminal branch of the closest RS, or the branch leading to the parental node of the LCA of the two closest RSs, depending on the strength of the signal of the closest RS. Notably, App-SpaM is able to provide both distal and pendant branch lengths for the placements it produces, and does so using an estimated phylogenetic distance (the Jukes-Cantor distance, Jukes and Cantor, 1969). Note that both APPLES and App-SpaM only produce a single placement per QS and can therefore not offer statistical measures of placement uncertainty such as the LWR.

Generally, distance-based placement methods produce results with lower accuracy compared to ML-based placement, though this gap appears to be narrowing. These newer approaches do however expand the scope of placement to sizes of reference trees, and lengths of reference sequences, that are orders of magnitude larger than what is currently possible with ML methods.

### 2.5 Application-Specific Placement Methods

Several additional placement methods exist. We provide a survey of these in this section. The placement methods covered in this section set themselves apart through their more specific use-cases, however this does not imply that their scope of use is necessarily limited.

#### 2.5.1 Viral Data

A particularly challenging use case for phylogenetic methods is the investigation of viral data, with a highly relevant example coming from the SARS-CoV-2 pandemic. Due to the dense sampling involved in studying such viral outbreaks, differences between individual taxa in a prospective tree may only be due to a very low number of, or even single, mutations. Consequently the amount of phylogenetic signal is generally very low, complicating tree reconstruction (Morel et al., 2020). Yet, distinguishing between major viral variants and identifying them precisely from a given clinical sample is crucial for epidemiological studies. In this context the UShER software was introduced that specifically focuses on phylogenetic placement of SARS-CoV-2 sequences (Turakhia et al., 2021). In contrast to ML methods, USHER uses a Maximum Parsimony (MP) approach, and does not operate on the full sequence alignment. This allows the method to focus directly on individual mutations, and consequently only use a fraction of the runtime and memory footprint of conventional ML placement methods. Note that the accuracy of MP-based phylogenetic methods can suffer when one or more lineages in the tree have experienced rapid evolution that results in long branch lengths. In such cases MP may incorrectly determine such lineages to be closely related, an effect termed *long branch attraction* (Felsenstein, 1978; Bergsten, 2005). While this is less of an issue for very closely related sequences such as SARS-CoV-2 or other (but not all) viral data, it may yield the application of such approaches to different types of data more challenging.

#### 2.5.2 Gene Trees

In principle, all placement methods aim to provide the location of a QS on a phylogeny that accurately reflects the underlying pattern of speciation, i. e., the *species tree*. In practice, the reference tree is typically only inferred on a single gene (16S, 18S, ITS, etc.), yielding a *gene tree* which may substantially differ from the species tree, called gene-tree *discordance* (Degnan and Rosenberg, 2009). Alternatively, we may have multiple such gene trees that induce a species tree, and subsequently want to perform query placement onto the species tree via placement onto the constituent gene trees (Sunagawa et al., 2013). Currently, only two placement methods are able to handle such cases: INSTRAL and DEPP. INSTRAL (Rabiee and Mirarab, 2019) performs placement of QSs for a species tree induced by a set of gene trees. It does so by first placing into the individual gene trees using existing ML placement methods, then re-inferring the species tree from the extended gene trees. In contrast to this, DEPP (Jiang et al., 2021, preprint) only considers the problem of discordance between a gene tree and its species tree and attempts to account for this during the placement into the species tree. The approach is based on a model of gene tree discordance learned from the data using deep neural networks that yields an embedding of given sequences into a euclidean space. Incidentally, this makes DEPP the first and so-far only phylogenetic placement method to incorporate machine learning. DEPP then uses the pairwise distances that result from the embedding of both reference and query sequences as input to APPLES, which computes the least-squares placement of the QSs.

#### 2.5.3 Other Use Cases

Some further tools make application-specific usage of placement. The first pertains to the specific case of samples containing sequences from exactly two organisms, and the task of identifying their respective known reference organisms. The tool MISA was developed with this specific use-case in mind (Balaban and Mirarab, 2020).

The second relates to either placing morphological sequences from fossils typically represented by binary characters (presence/absence of a trait) or Ancient DNA (aDNA) sequences. Placing ancient DNA sequences is generally challenging for analysis because of the high degree of degradation due to the age of the DNA molecules, generally shorter read lengths ranging between 50 and 150 base pairs, and post-mortem deamination (Hofreiter et al., 2001). The pathPhynder tool aims to solve this use-case (Martiniano et al., 2022, preprint). Like UShER, pathPhynder operates on nucleotide variants, focusing on single nucleotide polymorphisms. Furthermore, phylogenetic placement has been used for placement of fossils (Berger and Stamatakis, 2010; Bomfleur et al., 2015) using morphological data. This approach uses the maximum likelihood framework to use the signal from mixed morphological (binary) and molecular partitions in the underlying MSA.

Lastly, phylogenetic placement has also been proposed as a way to perform OTU clustering. The HmmUFOtu (Zheng et al., 2018) tool implements this specific use-case, along with automated taxonomic assignment (see also Section “Taxonomic Classification and Functional Analysis”). A unique characteristic in comparison to other placement tools is that HmmUFOtu also performs QS alignment and uses this information to pre-select promising placement locations.

### 2.6 Workflows Based on Phylogenetic Placement

Over the last decade, several pipelines have been published that use phylogenetic placement tools as their core method, building on it and using its result in various ways.

#### 2.6.1 Automated Analysis Pipelines

One class of placement pipelines focus on simplifying the overall use of placement methods, typically providing the user with the option to use a pre-computed reference tree, obviating the need for manual selection of reference taxa (Stark et al., 2010; Carbone et al., 2016; Douglas et al., 2018; Carbone et al., 2019; Douglas et al., 2020; Erazo et al., 2021; Sempéré et al., 2021). A number of these pipelines also automate the generation of key metrics and downstream analysis steps. Among these pipelines, of particular note is PICRUST2 (Douglas et al., 2018; Douglas et al., 2020), which stands out for accounting for 16S copy number correction, and providing the user with a prediction of the functional content of a sample. Similarly, paprica (Erazo et al., 2021) is a pipeline that computes metabolic pathway predictions for bacterial metagenomic sample data.

#### 2.6.2 Divide-And-Conquer Placement

A further key challenge for existing phylogenetic placement tools is scalability with regards to the size of the reference tree. While more recent methods have shown significant improvements in both the memory footprint and execution time required when placing QSs on reference trees on the order of 10^5^ reference taxa (see Section “Distance-Based Placement”), such input sizes remain extremely challenging for ML-based placement methods. A number of workflows have been proposed to scale existing placement methods for this use-case by splitting up the reference tree into smaller subtrees on which phylogenetic placement is then performed, creating a divide-and-conquer approach to phylogenetic placement (Mirarab et al., 2012; Czech et al., 2018; Czech et al., 2020; Koning et al., 2021; Wedell et al., 2021). These approaches vary primarily in how they select subtrees. SEPP (Mirarab et al., 2012) and pplacerDC (Koning et al., 2021) generate a subtree based on the topology of the reference tree. SEPP is a general boosting technique in particular for highly diverse reference trees (Liu et al., 2012; Mirarab et al., 2012). Further, a multi-level placement approach exists (Czech et al., 2018; Czech et al., 2020), which first places onto a broad RT, and then extracts QSs in pre-selected clades of that RT to place them again onto clade-specific high-resolution RTs. Finally, pplacer-XR (Wedell et al., 2021) selects a set of neighboring reference branches based on similarity to each query sequence, out of which it creates a subtree. Note that in this case, when decomposing the reference tree differently for every query sequence, scalability with regards to the number of query sequences is severely reduced.

A central promise of placement on very large trees is to simplify the curation and engineering tasks involved in creating a reference tree, as here a typical challenge is to decide which taxa to include in the tree. If placement can instead be performed on a tree encompassing an entire database, the curation challenge is circumvented. However, as another common issue with reference tree generation is the inclusion of overly similar reference sequences resulting in unclear or fuzzy placement signal, divide-and-conquer placement approaches may not be sufficient on their own.

#### 2.6.3 Evaluation of Placement Tools

Lastly, PEWO is an extensible testing framework specifically aimed at benchmarking and comparing different phylogenetic placement softwares (Linard et al., 2020). It includes a wide range of datasets and thus provides an important resource for identifying which placement tool is best suited for specific use-cases by evaluating the accuracy of existing tools, given some dataset. PEWO does so using a pruning-based evaluation procedure, where a subset of leaves is removed from a reference tree. This subset of sequences is subsequently used as input QSs for placement. The accuracy of a placement is calculated as the number of nodes between the best placement location, and the original location of the QS on the reference tree (called the node distance). This basic approach is used for evaluation in most publications that introduce new placement approaches. Note that the node distance measures two sources of error: error introduced by the placement algorithm, and error introduced by the pruning of the reference tree. In contrast to this, the “delta error” used in the evaluation of APPLES measures the additional error introduced through placement, in addition to the error introduced by the process of altering the reference tree through pruning (Metin et al., 2019). This new metric is however not yet included in the PEWO workflow. Nevertheless, the usefulness of a comprehensive and standardized testing framework cannot be emphasized enough, as it substantially facilitates further advancement and standardization in the field and the development of novel methods.

## 3 Visualization and Analysis

As mentioned before, there are two ways to conceptualize phylogenetic placement: 1) as an assignment (or mapping) of individual sequences to the branches of a phylogeny, usually taking the (*n*-)most likely placement location(s) of each sequence, or 2) as the distribution of all sequences of a sample across the tree, taking their respective abundances and placement probabilities into account. The former is similar to taxonomic assignment, but with full phylogenetic resolution instead of resolution at the taxonomic levels only, while the latter focuses on, e. g., species communities and their diversity as a whole. In the following we provide an overview of analysis methods that make use of such data.

### 3.1 Abundances and Multiplicities

In both interpretations, an important consideration is whether to take sequence abundances into account. When working with strictly identical sequences, or sequences resulting from some (OTU) clustering, the number of occurrences of each sequence or size of each cluster can be used as additional information for interpreting, e. g., community structure. On the one hand, including their abundances with the placement of each sequence yields information on how prevalent the species of these sequences are; for example, this can provide insight into the key (most abundant) species in environmental samples. On the other hand, dropping abundances and instead considering each sequence once (as a singleton) is more useful for estimating total diversity and taxonomic composition. For example, this way the number of *distinct* sequences can be regarded as a proxy for the number of species that are present in a sample. Whether to include abundances should hence be decided depending on the type of analysis conducted.

In the jplace format, these abundances can be stored as the so-called “multiplicity” of each placement (Matsen et al., 2012), in the "nm" data field. Unfortunately, the fasta (Pearson and Lipman, 1988) and phylip (Felsenstein, 1981) formats used as input to placement do not natively support abundance annotations, and current placement tools often do not handle them automatically, meaning that the information can be lost. However, the chunkify workflow (Czech et al., 2018; Czech et al., 2020) mentioned in Section “Clustering” takes abundances into account and annotates them as multiplicities in the resulting jplace file. Furthermore, gappa (Czech et al., 2020) offers a command to edit the multiplicities as needed, for example setting them post-hoc to the initial sequence abundance determination.

### 3.2 Visualization

Prior to more in-depth analyses, a first step in most workflows is a visualization of the immediate results. Following the two interpretations of phylogenetic placement (and hence, depending on the research question at hand), there are several ways to visualize placement results.

First, individual placements can be shown as actual branches attached to the RT, e. g., Figure 2C. Typically, only the most likely placement location per sequence is used for this, in order to avoid cluttering of the tree; this hence omits the information about uncertainty. This can be conducted by generating trees from placement results, e. g., in Newick format. Tools to this end are gappa (Czech et al., 2020) and guppy, which is part of pplacer (Matsen et al., 2010). This can subsequently be visualized via standard tree viewing tools (for a review, see Czech et al., 2019). Note however that such a visualizations can quickly become overloaded when the number of QSs becomes large.

Second, the LWR distribution of a single sequence can be visualized, to depict the uncertainty in placement across the tree, for example with ggtree (Yu et al., 2017) and ITOL (Letunic and Bork, 2016; Letunic and Bork, 2019).

**FIGURE 4:**
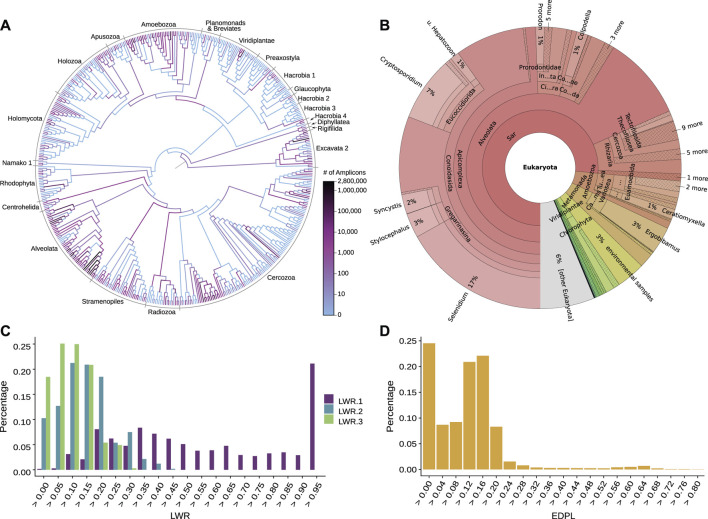
Examination of phylogenetic placement data. Here, we show some techniques for visually inspecting placement data, using an exemplary dataset consisting of 154 soil samples from neotropical rain forests placed on an eukaryotic reference tree (Mahé et al., 2017). **(A)** Heat tree showing the distribution of millions of amplicon reads on the reference tree by summing over the per-branch Likelihood Weight Ratios (LWRs) of all reads. The high abundance of placed reads in the *Alveolata* clade (dark branches in the lower left) visualizes a main finding of the dataset in form of an over-abundance of reads from that clade, shown in the phylogenetic context of the reference tree. Figure adapted from (Mahé et al., 2017). **(B)** Taxonomic assignment of all reads based on the PR^2^ (Benson et al., 2009; Guillou et al., 2012) taxonomy. The taxonomy of the reference sequences was used to label each branch of the reference tree by its highest non-conflicting taxonomic path. Then, for each read, the LWRs of its placement locations were accumulated for the branches, creating an overview of taxonomic abundances taking placement confidences into account. The result across all reads is shown here as a Krona plot (Ondov et al., 2011). **(C)** Histogram of the LWRs of the first three most likely placement locations of each read, showing how many of the reads have their first, second, and third most likely placement at each (binned) LWR value. For example, the highest bin of LWR.1 on the right hand side indicates that 20% of the reads have a first (most likely) placement position at or above an LWR of 0.95. That is, these placements have a high LWR and are hence placed with high certainty onto their respective branches. Note that the second most likely placement (LWR.2) can never have an LWR exceeding 1/2 (otherwise, it would be the most likely), the third most likely (LWR.3) not more than 1/3 (otherwise, it would be the second most likely), and so forth. **(D)** Histogram of the Expected Distance between Placement Locations (EDPL), which are computed as the distances (in terms of ML branch path length) between placement locations of a query sequence, weighted by the respective LWR of each location. The EDPL measures how far the placements of a sequence are spread across the branches of the reference tree, and hence how certain the placement in a “neighborhood” of the tree is. Here, most reads have an EDPL below 0.24 branch length units (mean expected number of substitutions per site). This indicates that the reads have most of their likely placements close to one another, within two branches on average, given that the used reference tree has an average branch length of about 0.12.

Third, the distribution of *all* sequences can be visualized directly on the reference tree, for example as shown in Figures 2E, 4A, taking their per-branch probabilities (and potentially their multiplicities/abundances) into account. This gives an overview of all placements, and can for example reveal important clades that received a high fraction of placements, or indicate whether placements are concentrated in a specific region of the tree. These visualizations can directly be generated by gappa (Czech et al., 2020) and iTOL (Letunic and Bork, 2016; Letunic and Bork, 2019); furthermore, guppy, can produce tree visualizations in the phyloXML format (Han and Zmasek, 2009), which can subsequently be displayed by tree viewer tools such as Archaeopteryx (Han and Zmasek, 2009).

### 3.3 Placement Quality and Uncertainty Quantification

An important post-analysis aspect is quality control, both in order to assess the suitability of the RT for the given placed sequences (to, e. g., test for missing reference sequences), and in order to assess the placed sequences themselves. Assuming a ‘perfect’ reference tree that exactly represents the diversity of the query sequences, the theoretical expectation is that each sequence gets placed onto a leaf of the tree with an LWR close to 1. Ignoring sequencing errors and other technical issues, deviations from this expectation can be due to several issues.

To this end, plotting the histograms or the distribution of the confidences (LWRs) across all placements can be useful, Figure 4C. A more involved metric is the so-called Expected Distance between Placement Locations (EDPL, Masten et al., 2010), which for a given sequence represents the uncertainty-weighted average distance between all placement locations of that sequence, or in other words, the sum of distances between locations, weighted by their respective probability, see Figure 4D. The EDPL is a measure of how far the likely placement locations of a sequence are spread out across the tree. It hence can distinguish between local and global uncertainty of the placements, that is, between cases where nearby edges constitute equally good placement locations versus cases where the sequence does not have a clear placement position in the tree (Matsen et al., 2010). These metrics can be explored with gappa (Czech et al., 2020) and guppy (Matsen et al., 2010); see their respective manuals for the available commands.

Examining the distribution of placement statistics, Figures 4C,D, or even the values of individual sequences, can help to identify the causes of problematic placements: 1) Sequences that are spread out across a clade with a flat placement distribution might indicate that too many closely related sequences, such as strains, are included in the RT; the EDPL can be used to quantify this. The query sequence is then likely another variant belonging to this subtree. 2) Placements towards inner branches of the RT might hint a hard to place query sequence, or at a lack of reference sequence diversity. This occurs if the (putative) ancestor represented by an inner node of the tree is more closely related to the QSs than the extant representatives included in the RT. This can either be the result of missing taxa in the RT, or even because the diversity of the clade is not fully known yet (also known as incomplete taxon sampling), in which case the QS might have originated from a previously undescribed species. 3) Sequences placed in two distinct clades might indicate technical errors such as the presence of chimeric sequences (Haas et al., 2011). 4) Sequences with elevated placement probability in multiple clades (e. g., placements in more than two subtrees) usually result from more severe issues, such as a total lack of suitable reference sequences for the QS, or a severe misalignment of the QS to the reference. This can for instance occur if metagenomic shotgun data has not been properly filtered, such that the genome region that the QS originated from is not included in the underlying MSA. 5) Lastly, long pendant lengths can also occur if a QS does not fit anywhere in the RT, in particular when the RT contains outgroups, which can cause long branch attraction for placed sequences (Bergsten, 2005).

Quantifying these uncertainties in a meaningful and interpretable way, and distinguishing between their causes, are open research questions. Approaches such as considering the EDPL, flatness of the LWR distribution, pendant lengths relative to the surrounding branch lengths of the RT, might help here, but more work is needed in order to distinguish actual issues from the identification of a new species based on their placement.

### 3.4 Taxonomic Classification and Functional Analysis

By understanding the taxonomic composition of an environment, questions about its species diversity and richness can be answered. Typical metagenomic data analyses hence often include a taxonomic classification of reads with respect to a database of known sequences (Breitwieser et al., 2019), for example by aggregating relative abundances per taxonomic group. In addition, such a classification based on known data enables to analyze which pathways and functions are present in a sample, and hence to gain insight into the metabolic capabilities of a microbial community.

#### 3.4.1 Preexisting Tools

Many tools exist to these ends: BLAST (Altschul et al., 1990) and other similarity-based methods were among the early methods, but depend on the threshold settings for various parameters (Shah et al., 2019), only provide meaningful results if the reference database contains sequences closely related to the queries (Mahé et al., 2017), and the closest hit does often not represent the most closely related species (Koski and Golding, 2001; Clemente et al., 2011). Thus, the advantages of leveraging the power of phylogenetics for taxonomic assignment have long been recognized (Delsuc and Ranwez, 2020). The classification can be based on *de novo* construction of a phylogeny (Krause et al., 2008; Schreiber et al., 2010), which as mentioned is computationally expensive, and tree topologies might change between samples, yielding downstream analyses and independent comparisons between studies challenging (Boyd et al., 2018). Other tools to investigate the community composition of metagenome datasets via phylogenomic assignment of markers genes are BUSCO (all kingdoms, Simão et al., 2015) and Amphora2 (Bacteria and Archaea, Wu and Scott, 2012). These allow relatively fast *de novo* phylogenetic search using several markers simultaneously. Alternatively, dedicated pipelines for 16S metabarcoding data such as QIIME (Caporaso et al., 2010; Bolyen et al., 2019) and mothur (Schloss et al., 2009) are routinely used to conduct taxonomic assignment based on sequence databases and established phylogenies as well as taxonomies; see Section “Sequence Selection” for a list of common databases, and see (López-García et al., 2018; Prodan et al., 2020) for comparisons of such pipelines. Other tools for taxonomic assignment and profiling are available, for example based on *k*-mers, which often use a fixed taxonomy such as the NCBI taxonomy (Benson et al., 2009; Sayers et al., 2009) to propose an evolutionary context for query sequences. They hence use a taxonomic tree without branch lengths, which can be an advantage when a fully resolved phylogeny is not available. Tools to this end are for example MEGAN (Huson et al., 2007), Kraken2 (Wood et al., 2014; Wood et al., 2019), and Kaiju (Menzel et al., 2016), see (Sczyrba et al., 2017; Bremges and McHardy, 2018; Meyer et al., 2019; Ye et al., 2019) for benchmarks and comparisons. However, these approaches are based on sequence similarity and related approaches, and can therefore be incongruent with the true underlying phylogenetic relationships of the sequences under comparison (Smith and Pease, 2017).

#### 3.4.2 Placement-Based Approaches

Phylogenetic placement can be employed to perform an accurate assignment of QSs to taxonomic labels (Czech et al., 2018), with potentially higher resolution than methods based on manually curated taxonomies (Darling et al., 2014; Rajter et al., 2021). This approach leverages models of sequence evolution (Darling et al., 2014), and is hence more accurate than similarity-based methods (von Mering et al., 2007). A further advantage over the above pipelines is the ability to use custom reference trees, thus providing a better context for interpreting the data under study. Incongruencies between the taxonomy and the phylogeny can however hinder the assignment, if they are not resolved (Matsen and Gallagher, 2012). Furthermore, it is important to note that placement-based methods only work when the query sequences are homologous to the available reference data, hence currently limiting the approach to, e. g., short genomes, metabarcoding or filtered metagenomic data.

A simple approach for taxonomic annotation based on placements is to label each branch of the RT by the most descriptive taxonomic path of its descendants, and to assign each QS to these labels based on its placement locations, potentially weighted by LWRs (Czech et al., 2018; Kozlov et al., 2016). This is implemented in gappa (Czech et al., 2020), see Figure 4B for an example; a similar visualization of the taxonomic assignment of placements can be conducted with BoSSA (Lefeuvre, 2018).

More involved and specialized approaches have also been suggested. PhyloSift (Darling et al., 2014) is a workflow that employs placement for taxonomic classification, using a database of gene families that are particularly well suited for metagenomics. The workflow further includes *Edge PCA* (introduced in Section “Similarity between Samples”) to assess community structure across samples, and offers Bayesian hypothesis testing for the presence of phylogenetic lineages. The gene-centric taxonomic profiling tool metAnnotate (Petrenko et al., 2015) uses a similar approach to identify organisms within a metagenomic sample that perform a function of interest. To this end, it searches shotgun sequences against the NCBI database (Benson et al., 2009; Sayers et al., 2009) first, and then employs placement to classify the reads with respect to genes and pathways of interest. GRAFTM (Boyd et al., 2018) is a tool for phylogenetic classification of genes of interest in large metagenomic datasets. Its primary application is to characterize sample composition using taxonomic marker genes, which can also target specific populations or functions. The abundance profiling methods TIPP (Nguyen et al., 2014) and TIPP2 (Shah et al., 2021) also use marker genes, and use the SEPP (Liu et al., 2012; Mirarab et al., 2012) boosting technique for phylogenetic placement with highly diverse reference trees, which increases classification accuracy when under-represented (novel) genomes are present in the dataset. The more recently introduced TreeSAPP tool (Morgan-Lang et al., 2020) uses a similar underlying framework, but improves functional and taxonomic annotation by regressing on the evolutionary distances (branch lengths) of the placed sequences, thereby increasing accuracy and reducing false discovery. Lastly, PHYLOMAGNET (Schön et al., 2019) is a workflow for gene-centric metagenome assembly (MAGs) that can determine the presence of taxa and pathways of interest in large short-read datasets. It allows to explore and pre-screen microbial datasets, in order to select good candidate sets for metagenomic assembly.

### 3.5 Diversity Estimates

A goal that is intrinsically connected to taxonomic assignment in studies that involve metagenomic and metabarcode sequencing is to quantify the diversity within a sample (called *α*-diversity) and the diversity between samples (called *β*-diversity). A plethora of methods exists to quantify the diversity of a set of sequences (for an excellent review, see Tucker et al., 2017). Here, we focus on those approaches that specifically work in conjunction with phylogenetic placement.

Among the *α*-diversity metrics, Faith’s Phylogenetic Diversity (PD) stands out, both for its widespread use in the literature and its direct use of phylogenetic information (Faith, 1992). More recently, a parameterized generalization of the PD was introduced that is able to interpolate between the classical PD and its abundance weighted formulation (McCoy and Matsen, 2013). Notably, this Balance Weighted Phylogenetic Diversity (BWPD) has been implemented to work directly with the results of phylogenetic placement, using the guppy fpd command (Matsen et al., 2010; Darling et al., 2014).

To our knowledge, the only other method that computes a measure of *α*-diversity directly from phylogenetic placement results is SCRAPP (Barbera et al., 2020), which also deploys species delimitation methods (Zhang et al., 2013; Kapli et al., 2017). In this method, the connection of phylogenetics to diversity is through the concept of a molecular species (Agapow et al., 2004), and quantifying how many such species are contained within a given sample. To facilitate this, SCRAPP resolves the between-QS phylogenetic relationships, resulting in per-reference-branch trees of those QSs that had their most likely placement on that specific branch. Thus, a byproduct of applying this method is a set of phylogenetic trees of the query sequences.

When the goal is to compute a *β*-diversity measure, a common choice for non-placement based approaches is the so-called Unifrac distance (Lozupone and Knight, 2005; Lozupone et al., 2007), which quantifies the relatedness of two communities that are represented by leaves of a shared phylogenetic tree. Interestingly, the weighted version of the Unifrac distance has been shown to be equivalent to the KR-distance (Evans and Matsen, 2012), see Section “Similarity between Samples”. As the Unifrac distance is widely used and well understood, this makes the KR-distance a safe choice for calculating between-sample distances, and thus a measure of *β*-diversity based on phylogenetic placement results.

### 3.6 Placement Distribution

Depending on the research question at hand, and for larger numbers of QSs, it is often more convenient and easier to interpret to look at the overall placement distribution instead of individually placed sequences. This distribution, as shown in Figures 2E, 4A, summarizes an entire sample (or even multiple samples) by adding up the per-branch probabilities (i. e., LWRs) of each placement location of all sequences in the sample(s), ignoring all branch lengths (distal, proximal, and pendant) of the placements. In this context, the accumulated per-branch probabilities are also called the *edge mass* of a given branch. This terminology is derived from viewing the reference tree as a graph consisting of nodes and edges, and viewing the placements as a mass distribution on that graph. This focuses more on the mathematical aspects of the data, and provides a useful framework for the analysis methods described below.

#### 3.6.1 Normalization of Absolute Abundances

High-throughput metagenomic sequence data are inherently compositional (Li, 2015; Gloor et al., 2017; Quinn et al., 2018), meaning that the total number of reads from HTS (absolute abundances) are mostly a function of available biological material and the specifics of the sequencing process. In other words, the total number of sequences per sample (often also called library size) is insignificant when comparing samples, see (Weiss et al., 2017; Du et al., 2018; Lin and Peddada, 2020) for reviews on this. This implies that sequence abundances are not comparable across samples, and that they can only be interpreted as proportions relative to each another (Calle, 2013; Silverman et al., 2017). However, the PCR amplification process is known to introduce biases (Logares et al., 2014), potentially skewing these proportions. For example, the relative abundances of the final amplicons do not necessarily reflect the original ratio of the input gene regions (Kanagawa, 2013; Li, 2015); this can be problematic in comparative studies. If these characteristics are not considered in analyses of the data (Weiss et al., 2017), spurious statistical results can occur (Aitchison, 1986; Jackson, 1997; Gloor et al., 2016; Tsilimigras and Fodor, 2016); see (Czech, 2020) for further details. For this reason, the estimation of indices such as the species richness is often implemented via so-called *rarefaction* and rarefaction curves (Gotelli and Colwell, 2001), which might however ignore a potentially large amount of the available valid data (McMurdie and Homes, 2014).

Phylogenetic placement of such data hence also needs to take this into account. The total edge masses (e. g., computed as the sum over all LWRs of a sample) are not informative, and merely reflect the total number of placed sequences. A simple strategy, upon which several of the analysis methods introduced below are based, is the normalization of the masses by dividing them by their total sum, effectively turning absolute abundances into relative abundances. This also eliminates the need for rarefaction, as low-abundance sequences only contribute marginally to the data. However, using this approach can still induce compositional artifacts in the data, as the per-branch probabilities (and hence the edge masses per sequence) have to sum to one for all branches of the tree. In other words, it is conceptually not possible to change the relative edge mass on a branch without also affecting edges masses on other branches.

#### 3.6.2 Transformations of Compositional Data

A statistically advantageous way to circumvent these effects, and resulting misinterpretations of compositional placement data, is to transform the data from per-branch values to per-clade values. This way, individual placement masses in the nearby branches of a clade are transformed into a single value for the entire clade, which expresses a measure of difference (called contrast) of the placement masses within the clade versus the masses in the remainder of the tree. This makes such transformations robust against placement uncertainty in a clade (e. g., due to similar reference sequences), implicitly captures the tree topology, and solves the issues of compositional data. From a technical point of view, this transforms the data from a compositional space into an Euclidean coordinate system (Juan and Pawlowsky-Glahn, 2005), where the individual dimensions of a data point are unconstrained and independent of each other. This can be achieved by utilizing the reference tree, whose branches imply bi-partitions of the two clades that are split by each branch (Pawlowsky-Glahn et al., 2015; Silverman et al., 2017). Instead of working with the per-branch placement masses, the accumulated masses on each side of a branch are contrasted against each other. This yields a view of the data that summarizes all placements in the clades implied by each branch. These transformations are, for example, achieved via two methods that in the existing literature have unfortunately confusingly similar names: imbalances and balances (Czech, 2020).

The edge *imbalance* (Matsen and Evans, 2013) is computed on the normalized edge masses of a sample: For each edge, the sum over all masses in the two clades defined by that edge are computed; their difference is then called the *imbalance* of the edge. The edge *balance* (Silverman et al., 2017; Czech and Stamatakis, 2019) is computationally similar, but instead of a difference of sums, it is computed as the (isometric) log-ratio of the geometric means of the masses in each clade; the resulting coordinates are called *balances* (Egozcue et al., 2003; Juan and Pawlowsky-Glahn, 2005; Quinn et al., 2018). Both transformations yield a contrast value for each (inner) branch of the tree, which can then, for example, be used to compare different samples to each other, see Section “Analysis of Multiple Samples”. They differ in the details of their statistical properties, but more work is needed to examine the effects of this on placement analyses (Czech, 2020); in practice, both can be (and are) used to avoid compositional artifacts. Alternatively, approaches such as Gamma-Poisson models and their zero-inflated versions (Peng et al., 2016; Weiss et al., 2017), as well as other methods for abundance normalization (Weiss et al., 2017; Du et al., 2018; Lin and Peddada, 2020) can be applied, although future work is needed to establish those in the context of phylogenetic placement.

### 3.7 Analysis of Multiple Samples

In typical metagenomic and metabarcoding studies, more than one sample is sequenced, e. g., from different locations or points in time of an environment. Furthermore, often per-sample metadata is collected as well, such as the pH-value of the soil or the temperature of the water where a sample was collected. These data allow to infer connections between the species community composition of the samples and environmental features. Given a set of samples (and potentially, metadata variables), an important goal is to understand the community structure (Tyson et al., 2020). To this end, fundamental tasks include measuring their similarity (a *distance* between samples), clustering samples that are similar to each other according to that distance measure, and relating the samples to their environmental variables. To this end, the methods introduced in this section utilize phylogenetic placement, and assume that the sequences from all samples have been placed onto the same underlying reference tree; they are implemented in gappa (Czech et al., 2020) and partially in guppy (Matsen et al., 2010).

#### 3.7.1 Similarity Between Samples

A simple first data exploration method consists in computing the *Edge Dispersion* (Czech and Stamatakis, 2019) of a set of samples, which detects branches or clades of the tree that exhibit a high heterogeneity across the samples by visualizing a measure of dispersion (such as the variance) of the per-sample placement mass. The method hence identifies branches and clades “of interest”, where samples differ in the amount of sequences being placed onto these parts of the tree.

The similarity between the placement distributions of two samples can be measured with the *phylogenetic Kantorovich-Rubinstein* (KR) distance (Evans and Matsen, 2012; Matsen and Evans, 2013), which is an adaptation of the Earth Mover’s distance to phylogenetic placement. The KR distance between two samples is a metric that quantifies by *at least* how much the normalized mass distribution of one sample has to be moved across the reference tree to obtain the distribution of the other sample. In other words, it is the minimum work needed to solve the transportation problem between the two distributions (transforming one into the other), and is related to the UniFrac distance (Lozupone and Knight, 2005; Lozupone et al., 2007). The distance is symmetrical, and increases the more mass needs to be moved (that is, the more the abundances per branch and clade differ between the two samples), and the larger the respective moving distance is (that is, the greater the phylogenetic distance along the branches of the tree between the clades is). It is hence an intuitive and phylogenetically informed distance metric for placement data, for example to quantify differences in the species composition of two environments.

**FIGURE 5:**
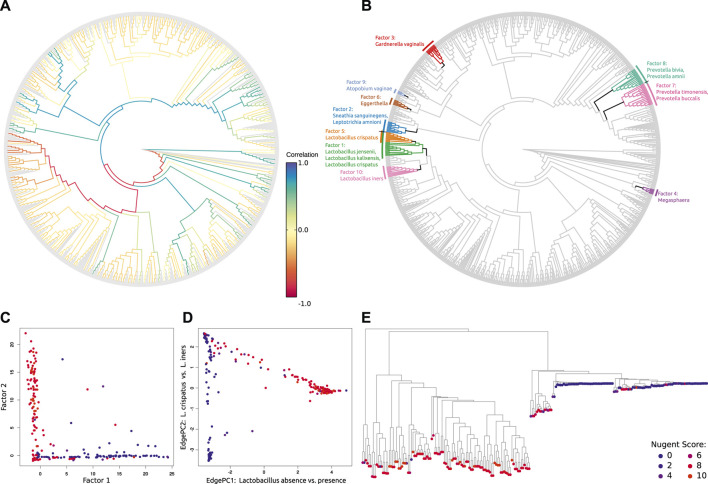
Analyses of phylogenetic placement data. Here, we show several analysis techniques for placement data, which relate multiple samples to each other (e. g., from different locations or points in time) that have been placed on the same underlying reference tree. The example dataset contains 220 vaginal samples of human patients with and without Bacterial Vaginosis (BV), a condition caused by an abnormal vaginal microbiome (Srinivasan et al., 2012), placed on a bacterial tree. The “Nugent” score is an external clinical indicator of the disease (Nugent et al., 1991), which is shown in **(C–E)** as blue (healthy, low score) vs. red colors (severe disease, high score). In healthy patients, two types of *Lactobacilli* dominate the microbiome, while in diseased patients, a diverse mixture of other bacteria take over. All figures are adapted from (Czech and Stamatakis, 2019), for details see (Srinivasan et al., 2012; Matsen and Evans, 2013; Czech and Stamatakis, 2019; Czech, 2020). **(A)** *Edge Correlation* between read abundances in clades of the reference tree (measured via the *imbalance* transformation) and the per-sample Nugent score. This visualization method identifies taxa whose abundances exhibit a relationship with environmental factors. Here, the red path towards the left identifies the *Lactobacillus* clade, that exhibits a strong anti-correlation with the Nugent score (healthy patients with a low score have high abundances in this clade), while blue and green paths show a multitude of clades that correlate with the score (diseased patients with a high score and high abundances in these diverse clades). **(B)** *Placement-Factorization* discretely identifies these clades by splitting up the tree into a number of “factors”: Black edges (with colorized clades below them) indicate the first ten factors (groups of taxa, some of them nested) whose differential placement abundances between samples exhibit a strong relationship with the Nugent score. That is, a factor is a clade in which abundances co-vary with metadata (e. g., the Nugent score). Here, these factors are again the *Lactobacillus* clade and a multitude of other clades that are also highlighted in **(A)** by colored paths. **(C)** *Placement-Factorization* can also ordinate samples, by plotting the *balances* (i. e., the abundance contrasts) across the edges identified by factors. Here, the first two factors of **(B)** are shown (each dot represents one sample, colored by its Nugent score), which split healthy and diseased patients. **(D)** *Edge Principal Components Analysis* (EdgePCA) is another ordination method, using PCA on the edge *imbalances*. Here, the first two PC axes are shown, which separate healthy from diseased patients (*Lactobacillus* presence vs. absence) on the first axis, and further distinguish the healthy patients based on the two types of *Lactobacilli* on the second axis. These interpretations of the axes are derived from visualizing the PCA directly on the reference tree, which is another way to show Edge PCA results, see (Matsen and Evans, 2013; Czech, 2020). **(E)** *Squash Clustering* is a hierarchical clustering method, here showing the clustering tree of the samples (not a phylogeny). Tip nodes (leaves) correspond to samples (individual patients), again colorized by their Nugent score, with samples clustered based on similarity of their placement distribution, and vertical distances showing this similarity, measured as the phylogenetic Kantorovich-Rubinstein (KR) distance between samples. Patients with a similar health status are close to each other, in particular the healthy (blue) ones.

*Edge Principal Component Analysis* (Edge PCA) is a method to detect community structure, which can also be employed for sample ordination and visualization (Darling et al., 2014; Matsen and Evans, 2013). Edge PCA identifies lineages of the RT that explain the greatest extent of variation between the sample communities, and is computed via standard Principal Component Analysis on the per-edge imbalances across all samples. The resulting principal components distinguish samples based on differences in abundances within clades of the reference tree. See for example Figure 5D, where each point corresponds to a sample and is colorized according to a metadata variable of the sample, showing that the ordination discriminates samples according to that variable. Furthermore, as the eigenvectors of each principal component correspond to edges of the tree, these can be visualized on the tree (Matsen and Evans, 2013; Czech, 2020), so that those edges and clades of the tree that explain differences between the samples can be identified, e. g., with guppy (Matsen et al., 2010) and Archaeopteryx (Han and Zmasek, 2009), or with gappa (Czech et al., 2020). Principal components can also be computed from the balances instead of the imbalances (Czech, 2020).

#### 3.7.2 Clustering of Samples

Given a measure of pairwise distance between samples, a fundamental task consists in clustering, that is, finding groups of samples that are similar according to that measure. *Squash Clustering* (Matsen and Evans, 2013) is a hierarchical agglomerative clustering method for a set of placement samples, and is based on the KR distance. Its results can be visualized as a clustering tree, where terminal nodes represent samples, each inner node represents the cumulative distribution of all samples below that node (“squashed” samples), and distances along the tree edges are KR distances. We show an example in Figure 5E, where each sample (terminal node) is colorized according to associated per-sample metadata variables (features measured for each sample), indicating that the clustering (based on the placement distribution) recovers characteristics of the samples based on that metadata variable.

The clustering hierarchy obtained from Squash Clustering grows with the number of samples, which contains a lot of detail, but can be cumbersome to visualize and interpret for large datasets with many samples. *Phylogenetic* *k* *-means* clustering and *Imbalance* *k* *-means* clustering (Czech and Stamatakis, 2019) are further clustering approaches, which instead yield an assignment of each sample to one of a predefined number of *k* clusters. Phylogenetic *k*-means uses the KR distance for determining the cluster assignment of the samples, and hence yields results that are consistent with Squash Clustering, while Imbalance *k*-means uses edge imbalances, and hence is consistent with results obtained from Edge PCA. Having the choice over the value *k* can be beneficial to answer specific questions with a known set of categories of samples (e. g., different body locations where samples were obtained from), but is also considered a downside of *k*-means clustering. Hence, various suggestions exist in the literature to select an appropriate *k* that reflects the number of “natural” clusters in the data (Thorndike, 1953; Rousseeuw, 1987; Bischof et al., 1999; Pelleg and Moore, 2000; Tibshirani et al., 2001; Hamerly et al., 2004). Visualizing the *cluster centroids* obtained from both methods can further help to interpret results by showing the average distributions of all samples in one of the *k* clusters; see again (Czech, 2020) for details.

#### 3.7.3 Relationship With Environmental Metadata Variables

The above methods only implicitly take metadata into account, e. g., by colorizing their resulting plots according to a variable. Environmental variables can also be incorporated explicitly in phylogenetic placement analysis, to more directly infer the relationships between the species composition of the samples (e. g., in form of abundances per clade) and the environments these communities live in.

The *Edge Correlation* (Czech and Stamatakis, 2019) visualizes parts of the tree where species abundances (as measured by the accumulated probability mass of each sample) exhibit a strong connection with a metadata variable, see Figure 5A. It is computed as the per-edge correlation coefficient between the per-sample metadata variable and either the edge masses (highlighting individual edges), or imbalances or balances (highlighting clades) of each sample.

*Placement-Factorization* (Czech and Stamatakis, 2019; Czech, 2020) is a more involved method. It is an adaption of *PhyloFactorization* (Washburne et al., 2017; Washburne et al., 2019) to phylogenetic placement data. Its goal is to identify branches in the tree along which putative functional traits might have arisen in adaptation to changes in environmental variables. In other words, it can detect clades of the reference tree whose abundances are linked to environmental factors. By “factoring out” the clade with the strongest signal in each step of the algorithm (hence the name of the method), nested dependencies with variables within clades can also be discovered, see Figure 5B. This factorization of the tree into nested clades can further be used as an ordination tool to visualize how samples are separated by changes along the factors, and as a dimensionality-reduction tool, see Figure 5C. The method assesses the relationship between per-sample metadata features and the balances computed on the samples; by using Generalized Linear Models, it allows to simultaneously incorporate multiple metadata variables of different types, such as numerical values (pH-value, temperature, latitude/longitude, etc), binary values (presence/absence patterns, diseased or not), or categorical values (body site that a sample was taken from).

## 4 Conclusion and Outlook

In this review we broadly surveyed the concepts, methods, and software tools that constitute and relate to phylogenetic placement. We have also presented guidelines and best practices for many typical use cases, showcased some common misconceptions and pitfalls, and introduced the most prominent downstream analysis methods. Phylogenetic placement is a versatile approach that is particularly applicable in metagenomics (e. g., for metabarcoding data) and broader eDNA-based ecology studies. It allows for the annotation of sequence data with phylogenetic information, and thereby to investigate the taxonomic content, functional capacity, diversity, and interactions of a community of organisms. Further, it allows for comparing samples from multiple spatial and temporal locations, enabling the analysis of community patterns across time and space, as well as their association with environmental metadata variables.

Despite the growing popularity of phylogenetic placement, there are several methodological and usage aspects that will benefit from further developments.

Currently, significant effort is required to create high-quality reference trees. We believe research effort should focus on simplifying this process, potentially through the design of methods that streamline and automate the commonly involved tasks. For example, while there are some metrics that quantify the quality of an inferred phylogenetic tree (Felsenstein, 1985; Dhar and Minin, 2016; Lemoine et al., 2018), there is a lack of metrics to specifically evaluate the suitability of a tree for phylogenetic placement, given some expected input data. Note that the PEWO testing framework (Linard et al., 2020) (see Section “Workflows based on Phylogenetic Placement”) represents a first step in this direction.

Ideally, reference trees and alignments should be created by, and shared in, research communities that investigate the same group(s) of organisms. This would not only yield obtaining high-quality reference trees trivial, but would also immensely increase the comparability across studies, as well as their reproducibility. Consequently, we would highly encourage such collaborations, and the public sharing of (perhaps even versioned instances of) gold-standard reference trees. Notably, for some environments, first efforts into this direction have already been undertaken (Berney et al., 2017; Del Campo et al., 2018; Rubinat-Ripoll, 2019; Rajter and Dunthorn, 2021; Rajter et al., 2021).

Furthermore, as mentioned, there is a lack of established methods that evaluate placement quality in a standardized and meaningful way. In particular, robust metrics are missing to distinguish the case where reference sequences of known species are missing from the tree from the case where the placed data actually contains yet undescribed species. A classification based on the LWR and pendant length of the placement locations might offer a solution here.

Lastly, further work is required to connect environmental metadata to the results of phylogenetic placement. Placement-based spatio-temporal methods are of high interest for addressing research questions in ecology and phylogeography. For example, relating geo-locations of samples to their placement could indicate how species communities differ across space, while creating placement time series could show how community compositions develop and change over time.

## References

[B1] Agapow P. M. Bininda-Emonds O. R. Crandall K. A. Gittleman J. L. Mace G. M. Marshall J. C. (2004). The Impact of Species Concept on Biodiversity Studies. Q. Rev. Biol. 79 (2), 161–179. 10.1086/383542 15232950

[B2] Aitchison J. (1986). The Statistical Analysis of Compositional Data. Chapman and Hall London.

[B3] Altschul S. F. Gish W. Miller W. Myers E. W. Lipman D. J. (1990). Basic Local Alignment Search Tool. J. Mol. Biol. 215 (3), 403–410. 10.1016/S0022-2836(05)80360-2 2231712

[B4] Amarasinghe S. L. Su S. Dong X. Zappia L. Ritchie M. E. Gouil Q. (2020). Opportunities and Challenges in Long-Read Sequencing Data Analysis. Genome Biol. 21 (1), 30. 10.1186/s13059-020-1935-5 32033565 PMC7006217

[B5] Angly F. E. Dennis P. G. Skarshewski A. Vanwonterghem I. Hugenholtz P. Tyson G. W. (2014). CopyRighter: a Rapid Tool for Improving the Accuracy of Microbial Community Profiles through Lineage-specific Gene Copy Number Correction. Microbiome 2 (1), 11. 10.1186/2049-2618-2-11 24708850 PMC4021573

[B98] Archie J. Day W. H. E. Maddison W. Meacham C. Rohlf F. J. Swofford D. (1986). The Newick Tree Format. Available at: http://evolution.genetics.washington.edu/phylip/newicktree.html .

[B6] Arenas M. (2015). Trends in Substitution Models of Molecular Evolution. Front. Genet. 6 (OCT), 319. 10.3389/fgene.2015.00319 26579193 PMC4620419

[B7] Auladell A. Sánchez P. Sánchez O. Gasol J. M. Ferrera I. (2019). Long-term Seasonal and Interannual Variability of marine Aerobic Anoxygenic Photoheterotrophic Bacteria. ISME J. 13 (138), 1975–1987. 10.1038/s41396-019-0401-4 30914777 PMC6776013

[B8] Balaban M. Mirarab S. (2020). Phylogenetic Double Placement of Mixed Samples. Bioinformatics 36, i335. 10.1093/bioinformatics/btaa489 32657414 PMC7355250

[B9] Balvočiūtė M. Huson D. H. (2017). SILVA, RDP, Greengenes, NCBI and OTT - How Do These Taxonomies Compare? BMC Genomics 18 (2), 114. 10.1186/s12864-017-3501-4 28361695 PMC5374703

[B10] Barbera P. Czech L. Lutteropp S. Stamatakis A. (2020). SCRAPP: A Tool to Assess the Diversity of Microbial Samples from Phylogenetic Placements. Mol. Ecol. Resour. 21 (1), 1755–0998. 10.1111/1755-0998.13255 PMC7756409 32996237

[B11] Barbera P. Kozlov A. M. Czech L. Morel B. Darriba D. Flouri T. (2018). Massively Parallel Evolutionary Placement of Genetic Sequences. Syst. Biol 68 (2), 365–369. 10.1093/sysbio/syy054 PMC6368480 30165689

[B12] Bartlett J. M. S. Stirling D. (2003). *A Short History Of the Polymerase Chain Reaction*. PCR Protocols. Methods Mol. Biol. 226, 3–6. 10.1385/1-59259-384-4:3 12958470

[B13] Bass D. Czech L. Williams B. A. P. Berney C. Dunthorn M. Mahé F. (2018). Clarifying the Relationships between Microsporidia and Cryptomycota. J. Eukaryot. Microbiol. 65 (6), 773–782. 10.1111/jeu.12519 29603494 PMC6282948

[B14] Beghini F. McIver L. J. Blanco-Míguez A. Dubois L. Asnicar F. Maharjan S. (2021). Integrating Taxonomic, Functional, and Strain-Level Profiling of Diverse Microbial Communities with bioBakery 3. eLife 10. 10.7554/elife.65088 PMC8096432 33944776

[B15] Benson D. A. Karsch-Mizrachi I. Lipman D. J. Ostell J. Sayers E. W. (2009). GenBank Nucleic Acids Res. 37, D26–D31. 10.1093/nar/gkn723 18940867 PMC2686462

[B16] Berger S. A. Stamatakis A. (2010). “Accuracy of Morphology-Based Phylogenetic Fossil Placement under Maximum Likelihood,” in ACS/IEEE International Conference on Computer Systems and Applications AICCSA. 10.1109/aiccsa.2010.5586939

[B17] Berger S. Stamatakis A. (2012). PaPaRa 2.0: A Vectorized Algorithm for Probabilistic Phylogeny-Aware Alignment Extension. Technical report. Heidelberg, Germany: Heidelberg Institute for Theoretical Studies, Heidelberg.

[B18] Berger S. A. Krompass D. Stamatakis A. (2011). Performance, Accuracy, and Web Server for Evolutionary Placement of Short Sequence Reads under Maximum Likelihood. Syst. Biol. 60 (3), 291–302. 10.1093/sysbio/syr010 21436105 PMC3078422

[B19] Berger S. A. Stamatakis A. (2011). Aligning Short Reads to Reference Alignments and Trees. Bioinformatics 27 (15), 2068–2075. 10.1093/bioinformatics/btr320 21636595

[B20] Bergsten J. (2005). A Review of Long-branch Attraction. Cladistics 21 (2), 163–193. 10.1111/j.1096-0031.2005.00059.x 34892859

[B21] Berney C. Ciuprina A. Bender S. Brodie J. Edgcomb V. Kim E. (2017). UniEuk: Time to Speak a Common Language in Protistology!. J. Eukaryot. Microbiol. 64 (1), 407–411. 10.1111/jeu.12414 28337822 PMC5435949

[B22] Bininda-Emonds O. R. Brady S. G. Kim J. Sanderson M. J. (2001). Scaling of Accuracy in Extremely Large Phylogenetic Trees. Pac. Symp. Biocomput 547–558, 547–558. 10.1142/9789814447362_0053 11262972

[B23] Bischof H. Leonardis A. Alexander S. (1999). MDL Principle for Robust Vector Quantisation. Pattern Anal. Appl. 2 (1), 59–72. 10.1007/s100440050015

[B24] Blanke M. Morgenstern B. (2021). App-SpaM: Phylogenetic Placement of Short Reads without Sequence Alignment. Bioinformatics Adv. 1 (1), 10. 10.1093/bioadv/vbab027 PMC9710606 36700102

[B25] Blaxter M. Mann J. Chapman T. Thomas F. Whitton C. Floyd R. (2005). Defining Operational Taxonomic Units Using DNA Barcode Data. Philos. Trans. R. Soc. Lond. B Biol. Sci. 360 (1462)–43. 10.1098/rstb.2005.1725 PMC1609233 16214751

[B26] Bolyen E. Rideout J. R. Dillon M. R. Bokulich N. A. Abnet C. C. Al-Ghalith G. A. (2019). Reproducible, Interactive, Scalable and Extensible Microbiome Data Science Using QIIME 2. Nat. Biotechnol. 37 (8), 852–857. 10.1038/s41587-019-0209-9 31341288 PMC7015180

[B27] Bomfleur B. Grimm G. W. McLoughlin S. (2015). Osmunda Pulchella Sp. Nov. From the Jurassic of Sweden-reconciling Molecular and Fossil Evidence in the Phylogeny of Modern Royal Ferns (Osmundaceae). BMC Evol. Biol. 15 (1), 1–25. 10.1186/s12862-015-0400-7 26123220 PMC4487210

[B28] Boyd J. A. Woodcroft B. J. Tyson G. W. (2018). GraftM: a Tool for Scalable, Phylogenetically Informed Classification of Genes within Metagenomes. Nucleic Acids Res. 46 (10), e59. 10.1093/nar/gky174 29562347 PMC6007438

[B239] Bray T. (2018). The JavaScript Object Notation (JSON) Data Interchange Format, RFC. Available at: https://tools.ietf.org/html/rfc7159 (Accessed August 14, 2018).

[B29] Brady A. Salzberg S. L. (2009). Phymm and PhymmBL: Metagenomic Phylogenetic Classification with Interpolated Markov Models. Nat. Methods 6 (9), 673–676. 10.1038/nmeth.1358 19648916 PMC2762791

[B30] Breitwieser F. P. Lu J. Salzberg S. L. (2019). A Review of Methods and Databases for Metagenomic Classification and Assembly. Brief Bioinform 20 (4), 1125–1136. 10.1093/bib/bbx120 29028872 PMC6781581

[B31] Bremges A. McHardy A. C. (2018). Critical Assessment of Metagenome Interpretation Enters the Second Round. mSystems 3 (4). 10.1128/mSystems.00103-18 PMC6040144 30003143

[B32] Brown D. G. Truszkowski J. (2012). “LSHPlace: Fast Phylogenetic Placement Using Locality-Sensitive Hashing,” in Biocomputing 2013 (World Scientific). 10.1142/9789814447973_0031 23424136

[B33] Callahan B. J. McMurdie P. J. Rosen M. J. Han A. W. Johnson A. J. Holmes S. P. (2016). DADA2: High-Resolution Sample Inference from Illumina Amplicon Data. Nat. Methods 13 (7), 581–583. 10.1038/nmeth.3869 27214047 PMC4927377

[B150] Calle M. L. (2013). Statistical Analysis of Metagenomics Data. Genomics Inform. 17 (1), e6. 10.5808/GI.2019.17.1.e6 PMC6459172 30929407

[B34] Caporaso J. G. Kuczynski J. Stombaugh J. Bittinger K. Costello E. K. Fierer N. (2010). QIIME Allows Analysis of High-Throughput Community Sequencing Data. Nat. Methods 7 (5), 335–336. 10.1038/nmeth0510-33510.1038/nmeth.f.303 20383131 PMC3156573

[B35] Carbone I. White J. B. Miadlikowska J. Arnold A. E. Miller M. A. Magain N. (2019). T-BAS Version 2.1: Tree-Based Alignment Selector Toolkit for Evolutionary Placement of DNA Sequences and Viewing Alignments and Specimen Metadata on Curated and Custom Trees. Microbiol. Resour. Announc 8 (29). 10.1128/mra.00328-19 PMC6639605 31320426

[B36] Carbone I. White J. B. Miadlikowska J. Arnold A. E. Miller M. A. Kauff F. (2016). T-BAS: Tree-Based Alignment Selector Toolkit for Phylogenetic-Based Placement, Alignment Downloads and Metadata Visualization: an Example with the Pezizomycotina Tree of Life. Bioinformatics, btw808. 10.1093/bioinformatics/btw808 28003260

[B37] Cardoni S. Piredda R. Denk T. Grimm G. W. Papageorgiou A. C. Schulze E. D. (2022). 5S-IGS rDNA in Wind-Pollinated Trees (Fagus L.) Encapsulates 55 Million Years of Reticulate Evolution and Hybrid Origins of Modern Species. Plant J. 109 (4), 909–926. 10.1111/tpj.15601 34808015 PMC9299691

[B38] Chatzou M. Magis C. Chang J. M. Kemena C. Bussotti G. Erb I. (2016). Multiple Sequence Alignment Modeling: Methods and Applications. 10.1093/bib/bbv099 26615024

[B39] Clare E. L. Economou C. K. Bennett F. J. Dyer C. E. Adams K. McRobie B. (2022). Measuring Biodiversity from DNA in the Air. Curr. Biol. 32, 693–700. 10.1016/j.cub.2021.11.064 34995488

[B40] Clemente J. C. Jansson J. Valiente G. (2011). Flexible Taxonomic Assignment of Ambiguous Sequencing Reads. BMC Bioinformatics 12 (1), 8–15. 10.1186/1471-2105-12-8 21211059 PMC3024944

[B41] Cole J. R. Wang Q. Fish J. A. Chai B. McGarrell D. M. Sun Y. (2014). Ribosomal Database Project: Data and Tools for High Throughput rRNA Analysis. Nucleic Acids Res. 42, D633–D642. 10.1093/nar/gkt1244 24288368 PMC3965039

[B42] Collins R. A. Trauzzi G. Maltby K. M. Gibson T. I. Ratcliffe F. C. Hallam J. (2021). Meta‐Fish‐Lib : A Generalised, Dynamic DNA Reference Library Pipeline for Metabarcoding of Fishes. J. Fish Biol. 99 (4), 1446–1454. 10.1111/jfb.14852 34269417

[B43] Curtis H. Gevers D. Knight R. Abubucker S. Badger J. H. Chinwalla A. T. (2012). Structure, Function and Diversity of the Healthy Human Microbiome. Nature 486 (7402), 207–214. 10.1038/nature11234 22699609 PMC3564958

[B44] Czech L. Barbera P. Stamatakis A. (2020). Genesis and Gappa: Processing, Analyzing and Visualizing Phylogenetic (Placement) Data. Bioinformatics 36 (10), 3263–3265. 10.1093/bioinformatics/btaa070 32016344 PMC7214027

[B45] Czech L. Barbera P. Stamatakis A. (2018). Methods for Automatic Reference Trees and Multilevel Phylogenetic Placement. Bioinformatics 35 (7), 1151–1158. 10.1093/bioinformatics/bty767 PMC6449752 30169747

[B46] Czech L. Huerta-Cepas J. Stamatakis A. (2019). A Critical Review on the Use of Support Values in Tree Viewers and Bioinformatics Toolkits. Mol. Biol. Evol. 17 (4), 383–384. 10.1093/molbev/msx055 PMC5435079 28369572

[B47] Czech L. Stamatakis A. (2019). Scalable Methods for Analyzing and Visualizing Phylogenetic Placement of Metagenomic Samples. PLOS ONE 14 (5), e0217050. 10.1371/journal.pone.0217050 31136592 PMC6538146

[B149] Czech L. (2020). Novel Methods for Analyzing and Visualizing Phylogenetic Placements. Ph.D. thesis. Karlsruhe, Germany: Karlsruher Institut für Technologie. 10.5445/IR/1000105237

[B50] Darling A. E. Jospin G. Lowe E. Matsen F. A. Bik H. M. Eisen J. A. (2014). PhyloSift: Phylogenetic Analysis of Genomes and Metagenomes. PeerJ 2, e243. 10.7717/peerj.243 24482762 PMC3897386

[B51] Degnan J. H. Rosenberg N. A. (2009). Gene Tree Discordance, Phylogenetic Inference and the Multispecies Coalescent. Trends Ecol. Evol. 24 (6), 332–340. 10.1016/j.tree.2009.01.009 19307040

[B52] Deiner K. Bik H. M. Mächler E. Seymour M. Lacoursière-Roussel A. Altermatt F. (2017). Environmental DNA Metabarcoding: Transforming How We Survey Animal and Plant Communities. Mol. Ecol. 26 (21), 5872–5895. 10.1111/mec.14350 28921802

[B53] Del Campo J. Kolisko M. Boscaro V. Santoferrara L. F. Nenarokov S. Massana R. (2018). EukRef: Phylogenetic Curation of Ribosomal RNA to Enhance Understanding of Eukaryotic Diversity and Distribution. Plos Biol. 16 (9), e2005849–14. 10.1371/journal.pbio.2005849 30222734 PMC6160240

[B54] Delsuc F. Ranwez V. (2020). “Accurate Alignment of (Meta)barcoding Data Sets Using MACSE,” in Phylogenetics in the Genomic Era. Editors Scornavacca C. Delsuc F. Galtier N.. Available at: https://hal.archives-ouvertes.fr/hal-02541199 .

[B55] Desai N. Antonopoulos D. Gilbert J. A. Glass E. M. Meyer F. (2012). From Genomics to Metagenomics. Curr. Opin. Biotechnol. 23 (1), 72–76. 10.1016/j.copbio.2011.12.017 22227326

[B56] DeSantis T. Z. Hugenholtz P. Larsen N. Rojas M. Brodie E. L. Keller K. (2006). Greengenes, a Chimera-Checked 16S rRNA Gene Database and Workbench Compatible with ARB. Appl. Environ. Microbiol. 72 (7), 5069–5072. 10.1128/AEM.03006-05 16820507 PMC1489311

[B57] Dhar A. Minin V. N. (2016). Maximum Likelihood Phylogenetic Inference. Encyclopedia Evol. Biol. 2, 499–506. 10.1016/b978-0-12-800049-6.00207-9

[B58] Dodsworth S. (2015). Genome Skimming for Next-Generation Biodiversity Analysis. Trends Plant Sci. 20 (9), 525–527. 10.1016/j.tplants.2015.06.012 26205170

[B59] Douglas C. (2018). The Application/json Media Type for JavaScript Object Notation (JSON), RFC. Available at: https://tools.ietf.org/html/rfc4627 (Accessed August 14, 2018).

[B60] Douglas G. M. Maffei V. J. Zaneveld J. R. Yurgel S. N. Brown J. R. Taylor C. M. (2020). PICRUSt2 for Prediction of Metagenome Functions. Nat. Biotechnol., 1–5. 10.1038/s41587-020-0548-6 32483366 PMC7365738

[B61] Douglas G. M. Beiko R. G. Langille M. G. I. (2018). “Predicting the Functional Potential of the Microbiome from Marker Genes Using PICRUSt,” in Microbiome Analysis (Springer), 169–177. 10.1007/978-1-4939-8728-3_11 30298254

[B62] Du R. An L. Fang Z. (2018). Performance Evaluation of Normalization Approaches for Metagenomic Compositional Data on Differential Abundance Analysis. Cham: Springer International Publishing, 329–344. 10.1007/978-3-319-99389-8_16

[B63] Dunthorn M. Otto J. Berger S. A. Stamatakis A. Mahé F. Romac S. (2014). Placing Environmental Next-Generation Sequencing Amplicons from Microbial Eukaryotes into a Phylogenetic Context. Mol. Biol. Evol. 31 (4), 993–1009. 10.1093/molbev/msu055 24473288

[B64] Dupont A. Ö. Griffiths R. I. Bell T. Bass. D. (2016). Differences in Soil Micro-eukaryotic Communities over Soil pH Gradients Are Strongly Driven by Parasites and Saprotrophs. Environ. Microbiol. 18 (6), 2010–2024. 10.1111/1462-2920.13220 26768496

[B218] Eddy S. R. (1995). Multiple Alignment Using Hidden Markov Models. Proc. Int. Conf. Intell. Syst. Mol. Biol. 3, 114–120. 7584426

[B65] Eddy S. R. (1998). Profile Hidden Markov Models. Bioinformatics 14 (9), 755–763. 10.1093/bioinformatics/14.9.755 9918945

[B66] Edgar R. C. (2021). MUSCLE V5 Enables Improved Estimates of Phylogenetic Tree Confidence by Ensemble Bootstrapping. bioRxiv. 10.1101/2021.06.20.449169

[B67] Edgar R. C. (2004). MUSCLE: Multiple Sequence Alignment with High Accuracy and High Throughput. Nucleic Acids Res. 32 (5), 1792–1797. 10.1093/nar/gkh340 15034147 PMC390337

[B68] Edgar R. C. (2010). Search and Clustering Orders of Magnitude Faster Than BLAST. Bioinformatics 26 (19), 2460–2461. 10.1093/bioinformatics/btq461 20709691

[B69] Edwards D. J. Holt K. E. (2013). Beginner's Guide to Comparative Bacterial Genome Analysis Using Next-Generation Sequence Data. Microb. Inform. Exp. 3 (1), 2. 10.1186/2042-5783-3-2 23575213 PMC3630013

[B70] Egozcue J. J. Pawlowsky-Glahn V. Mateu-Figueras G. Barceló-Vidal C. (2003). Isometric Logratio Transformations for Compositional Data Analysis. Math. Geology. 35 (3), 279–300. 10.1023/A:1023818214614

[B71] ElRakaiby M. T. Gamal-Eldin S. Amin M. A. Aziz R. K. (2019). Hospital Microbiome Variations as Analyzed by High-Throughput Sequencing. OMICS 23 (9), 426–438. 10.1089/omi.2019.0111 31393213

[B72] Erazo N. G. Dutta A. Bowman J. S. (2021). From Microbial Community Structure to Metabolic Inference Using Paprica. STAR Protoc. 2 (4), 101005. 10.1016/j.xpro.2021.101005 34950886 PMC8672035

[B73] Escobar-Zepeda A. Vera-Ponce De León A. Sanchez-Flores A. (2015). The Road to Metagenomics: From Microbiology to DNA Sequencing Technologies and Bioinformatics. Front. Genet. 6 (348), 1–15. 10.3389/fgene.2015.00348 26734060 PMC4681832

[B74] Evans S. N. Matsen F. A. (2012). The Phylogenetic Kantorovich-Rubinstein Metric for Environmental Sequence Samples. J. R. Stat. Soc. Ser. B Stat Methodol 74, 569–592. 10.1111/j.1467-9868.2011.01018.x PMC3405733 22844205

[B49] Faith P. D. (1992). Conservation Evaluation and Phylogenetic Diversity. Biol. Conservation 61 (1), 1–10. 10.1016/0006-3207(92)91201-3

[B104] Felsenstein J. (1978). Cases in Which Parsimony or Compatibility Methods Will Be Positively Misleading. Syst. Biol. 27 (4), 401–410. 10.1093/sysbio/27.4.401

[B75] Felsenstein J. (1981). Evolutionary Trees from DNA Sequences: A Maximum Likelihood Approach. J. Mol. Evol. 17 (6), 368–376. 10.1007/BF01734359 7288891

[B105] Felsenstein J. (1985). Confidence Limits on Phylogenies: an Approach Using the Bootstrap. Evolution 39 (4), 783–791. 10.1111/j.1558-5646.1985.tb00420.x 28561359

[B106] Felsenstein J. (2004). Inferring Phylogenies. 2nd edition. MA: Sinauer Associates Sunderland. 978-0878931774.

[B76] Fu L. Niu B. Zhu Z. Wu S. Li W. (2012). CD-HIT: Accelerated for Clustering the Next-Generation Sequencing Data. Bioinformatics 28 (23), 3150–3152. 10.1093/bioinformatics/bts565 23060610 PMC3516142

[B77] Giner C. R. Forn I. Romac S. Logares R. De Vargas C. Massana R. (2016). Environmental Sequencing Provides Reasonable Estimates of the Relative Abundance of Specific Picoeukaryotes. Appl. Environ. Microbiol. 82 (15), 4757–4766. 10.1128/AEM.00560-16 27235440 PMC4984273

[B78] Gloor G. B. Macklaim J. M. Vu M. Fernandes A. D. (2016). Compositional Uncertainty Should Not Be Ignored in High-Throughput Sequencing Data Analysis. Austrian J. Stat. 45 (4), 73. 10.17713/ajs.v45i4.122

[B79] Gloor G. B. Macklaim J. M. Pawlowsky-Glahn V. Egozcue J. J. (2017). Microbiome Datasets Are Compositional: And This Is Not Optional. Front. Microbiol. 8, 2224. 10.3389/fmicb.2017.02224 29187837 PMC5695134

[B80] Gohli J. Bøifot K. O. Moen L. V. Pastuszek P. Skogan G. Udekwu K. I. (2019). The Subway Microbiome: Seasonal Dynamics and Direct Comparison of Air and Surface Bacterial Communities. Microbiome 7 (1), 1–16. 10.1186/s40168-019-0772-9 31856911 PMC6924074

[B81] Goodwin S. McPherson J. D. McCombie W. R. (2016). Coming of Age: Ten Years of Next-Generation Sequencing Technologies. Nat. Rev. Genet. 17 (6), 333–351. 10.1038/nrg.2016.49 27184599 PMC10373632

[B82] Gotelli N. J. Colwell R. K. (2001). Quantifying Biodiversity: Procedures and Pitfalls in the Measurement and Comparison of Species Richness. Ecol. Lett. 4 (4), 379–391. 10.1046/j.1461-0248.2001.00230.x

[B83] Guillou L. Bachar D. Audic S. Bass D. Berney C. Bittner L. (2012). The Protist Ribosomal Reference Database (PR2): a Catalog of Unicellular Eukaryote Small Sub-unit rRNA Sequences with Curated Taxonomy. Nucleic Acids Res. 41 (D1), D597–D604. 10.1093/nar/gks1160 23193267 PMC3531120

[B84] Haas B. J. Gevers D. Earl A. M. Ward V. Giannoukos G. Ciulla D. (2011). Chimeric 16S rRNA Sequence Formation and Detection in Sanger and 454-pyrosequenced PCR Amplicons. Genome Res. 21 (3), 494–504. 10.1101/gr.112730.110 21212162 PMC3044863

[B85] Hamerly G. Elkan C. (2004). “Learning the K in K-Means,” in Advances in Neural Information Processing Systems. Editors Thrun S. Saul L. K. Schölkopf P. B. (MIT Press), 16, 281–288.

[B86] Han M. V. Zmasek C. M. (2009). phyloXML: XML for Evolutionary Biology and Comparative Genomics. BMC Bioinformatics 10, 356. 10.1186/1471-2105-10-356 19860910 PMC2774328

[B87] Hanson B. Zhou Y. Bautista E. J. Urch B. Speck M. Silverman F. (2016). Characterization of the Bacterial and Fungal Microbiome in Indoor Dust and Outdoor Air Samples: a Pilot Study. Environ. Sci. Process. Impacts 18 (6), 713–724. 10.1039/c5em00639b 27213188 PMC5015483

[B88] Heather J. M. Chain B. (2016). The Sequence of Sequencers: The History of Sequencing DNA. Genomics 107 (1), 1–8. 10.1016/j.ygeno.2015.11.003 26554401 PMC4727787

[B89] Hebert P. D. Cywinska A. Ball S. L. deWaard J. R. (2003). Biological Identifications through DNA Barcodes. Proc. Biol. Sci. 270 (1512), 313–321. 10.1098/rspb.2002.2218 12614582 PMC1691236

[B90] Hleap J. S. Littlefair J. E. Steinke D. Hebert P. D. N. Cristescu M. E. (2021). Assessment of Current Taxonomic Assignment Strategies for Metabarcoding Eukaryotes. Mol. Ecol. Resour. 21 (7), 2190–2203. 10.1111/1755-0998.13407 33905615

[B91] Hofreiter M. Serre D. Poinar H. N. Kuch M. Pääbo S. Ancient D. N. A. (2001). Ancient DNA. Nat. Rev. Genet. 2 (5), 353–359. 10.1038/35072071 11331901

[B92] Holder M. Lewis P. O. (2003). Phylogeny Estimation: Traditional and Bayesian Approaches. Nat. Rev. Genet. 4 (4), 275–284. 10.1038/nrg1044 12671658

[B93] Hubert F. Grimm G. W. Jousselin E. Berry V. Franc A. Kremer A. (2014). Multiple Nuclear Genes Stabilize the Phylogenetic Backbone of the genusQuercus. Syst. Biodiversity 12 (4), 405–423. 10.1080/14772000.2014.941037

[B94] Huelsenbeck J. P. Ronquist F. Nielsen R. Bollback J. P. (5550). Bayesian Inference of Phylogeny and its Impact on Evolutionary Biology. Science 294, 2310–2314. 10.1126/science.1065889 11743192

[B95] Hugerth L. W. Andersson A. F. (2017). Analysing Microbial Community Composition through Amplicon Sequencing: From Sampling to Hypothesis Testing. Front. Microbiol. 8, 1561. 10.3389/fmicb.2017.01561 28928718 PMC5591341

[B96] Huson D. H. Auch A. F. Qi J. Schuster S. C. (2007). MEGAN Analysis of Metagenomic Data. Genome Res. 17 (3), 377–386. 10.1101/gr.5969107 17255551 PMC1800929

[B97] Jackson D. A. (1997). Compositional Data in Community Ecology: The Paradigm or Peril of Proportions? Ecology 78 (3), 929–940. 10.1890/0012-9658(1997)078[0929:cdicet]2.0.co;2

[B99] Jamy M. Foster R. Barbera P. Czech L. Kozlov A. Stamatakis A. (2019). Long-read Metabarcoding of the Eukaryotic rDNA Operon to Phylogenetically and Taxonomically Resolve Environmental Diversity. Mol. Ecol. Resour. 20 (2), 429–443. 10.1111/1755-0998.13117 31705734

[B100] Janssen S. McDonald D. Gonzalez A. Navas-Molina J. A. Jiang L. Xu Z. Z. (2018). Phylogenetic Placement of Exact Amplicon Sequences Improves Associations with Clinical Information. mSystems 3 (3), e00021–18. 10.1128/mSystems.00021-18 29719869 PMC5904434

[B101] Jeong J. Yun K. Mun S. Chung W.-H. Choi S.-Y. Nam Y.-d. (2021). The Effect of Taxonomic Classification by Full-Length 16s rRNA Sequencing with a Synthetic Long-Read Technology. Sci. Rep. 11 (1), January. 10.1038/s41598-020-80826-9 PMC7814050 33462291

[B102] Ji Y. Ashton L. Pedley S. M. Edwards D. P. Tang Y. Nakamura A. (2013). Reliable, Verifiable and Efficient Monitoring of Biodiversity via Metabarcoding. Ecol. Lett. 16 (10), 1245–1257. 10.1111/ele.12162 23910579

[B103] Jiang Y. Metin B. Zhu Q. Mirarab S. (2021). DEPP: Deep Learning Enables Extending Species Trees Using Single Genes. bioRxiv. 10.1101/2021.01.22.427808 PMC10198656 35485976

[B107] Juan J. E. Pawlowsky-Glahn V. (2005). Groups of Parts and Their Balances in Compositional Data Analysis. Math. Geology. 37 (7), 795–828. 10.1007/s11004-005-7373-9

[B235] Jukes T. H. Cantor C. R. (1969). Mammalian Protein Metabolism. Chapter Evolution of protein molecules. New York, United States: Academic Press, Inc. 3, 21–132.

[B108] Kanagawa T. (2013). Bias and Artifacts in Multitemplate Polymerase Chain Reactions (PCR). J. Biosci. Bioeng. 96 (4), 317–323. 10.1016/S1389-1723(03)90130-7 16233530

[B109] Kapli P. Lutteropp S. Zhang J. Kobert K. Pavlidis P. Stamatakis A. (2017). Multi-rate Poisson Tree Processes for Single-Locus Species Delimitation under Maximum Likelihood and Markov Chain Monte Carlo. Bioinformatics 33 (11), 1630–1638. 10.1093/bioinformatics/btx025 28108445 PMC5447239

[B110] Kapli P. Yang Z. Telford M. J. (2020). Phylogenetic Tree Building in the Genomic Age. Nat. Rev. Genet. 21 (7), 428–444. 10.1038/s41576-020-0233-0 32424311

[B111] Karsenti E. Acinas S. G. Bork P. Bowler C. De Vargas C. Raes J. (2011). A Holistic Approach to marine Eco-Systems Biology. Plos Biol. 9 (10), e1001177–11. 10.1371/journal.pbio.1001177 22028628 PMC3196472

[B112] Katoh K. Frith M. C. (2012). Adding Unaligned Sequences into an Existing Alignment Using MAFFT and LAST. Bioinformatics 28 (23), 3144–3146. 10.1093/bioinformatics/bts578 23023983 PMC3516148

[B113] Katoh K. Misawa K. Kuma K. Miyata T. (2002). MAFFT: a Novel Method for Rapid Multiple Sequence Alignment Based on Fast Fourier Transform. Nucleic Acids Res. 30 (14), 3059–3066. 10.1093/nar/gkf436 12136088 PMC135756

[B114] Katz K. Shutov O. Lapoint R. Kimelman M. Brister J. R. O'Sullivan C. (2022). The Sequence Read Archive: a Decade More of Explosive Growth. Nucleic Acids Res. 50 (D1), D387–D390. 10.1093/nar/gkab1053 34850094 PMC8728234

[B115] Keck F. Vasselon V. Rimet F. Bouchez A. Kahlert M. (2018). Boosting DNA Metabarcoding for Biomonitoring with Phylogenetic Estimation of Operational Taxonomic Units' Ecological Profiles. Mol. Ecol. Resour. 18 (6), 1299–1309. 10.1111/1755-0998.12919 29923321

[B116] Kembel S. W. Wu M. Eisen J. A. Green J. L. (2012). Incorporating 16s Gene Copy Number Information Improves Estimates of Microbial Diversity and Abundance. Plos Comput. Biol. 8 (10), e1002743. 10.1371/journal.pcbi.1002743 23133348 PMC3486904

[B117] Kemena C. Notredame C. (2009). Upcoming Challenges for Multiple Sequence Alignment Methods in the High-Throughput Era. Bioinformatics 25 (19), 2455–2465. 10.1093/bioinformatics/btp452 19648142 PMC2752613

[B118] Koning E. Phillips M. Warnow T. (2021). “pplacerDC: a New Scalable Phylogenetic Placement Method” in Proceedings of the 12th ACM Conference on Bioinformatics (Gainesville, Florida: Computational Biology, and Health Informatics), 1–9.

[B119] Koski L. B. Golding G. B. (2001). The Closest BLAST Hit Is Often Not the Nearest Neighbor. J. Mol. Evol. 52 (6), 540–542. 10.1007/s002390010184 11443357

[B120] Kozlov A. M. Darriba D. Flouri T. Morel B. Stamatakis A. (2019). A Fast, Scalable, and User-Friendly Tool for Maximum Likelihood Phylogenetic Inference Bioinformatics 35 (21), 4453–4455. 10.1093/bioinformatics/btz305 31070718 PMC6821337

[B121] Kozlov A. M. Zhang J. Yilmaz P. Glöckner F. O. Stamatakis A. (2016). Phylogeny-aware Identification and Correction of Taxonomically Mislabeled Sequences. Nucleic Acids Res. 44 (11), 5022–5033. 10.1093/nar/gkw396 27166378 PMC4914121

[B122] Krause L. Diaz N. N. Goesmann A. Kelley S. Nattkemper T. W. Rohwer F. (2008). Phylogenetic Classification of Short Environmental DNA Fragments. Nucleic Acids Res. 36 (7), 2230–2239. 10.1093/nar/gkn038 18285365 PMC2367736

[B123] Kress W. J. Erickson D. L. (2008). DNA Barcodes: Genes, Genomics, and Bioinformatics. Proc. Natl. Acad. Sci. U S A. 105 (8), 2761–2762. 10.1073/pnas.0800476105 18287050 PMC2268532

[B124] Kuleshov V. Jiang C. Zhou W. Jahanbani F. Batzoglou S. Snyder M. (2016). Synthetic Long-Read Sequencing Reveals Intraspecies Diversity in the Human Microbiome. Nat. Biotechnol. 34 (1), 64–69. 10.1038/nbt.3416 26655498 PMC4884093

[B125] Lacoursière-Roussel A. Côté G. Leclerc V. Bernatchez L. (2016). Quantifying Relative Fish Abundance with eDNA: a Promising Tool for Fisheries Management. J. Appl. Ecol. 53 (4), 1148–1157. 10.1111/1365-2664.12598

[B126] Langmead B. Salzberg S. L. (2012). Fast Gapped-Read Alignment with Bowtie 2. Nat. Methods 9 (4), 357–359. 10.1038/nmeth.1923 22388286 PMC3322381

[B127] Lee Z. M. Bussema C. Schmidt T. M. (2009). rrnDB: Documenting the Number of rRNA and tRNA Genes in Bacteria and Archaea. Nucleic Acids Res. 37, D489–D493. 10.1093/nar/gkn689 18948294 PMC2686494

[B128] Lefeuvre P. (2018). BoSSA: A Bunch of Structure and Sequence Analysis.

[B129] Lemoine F. Domelevo Entfellner J.-B. Wilkinson E. Correia D. Dávila Felipe M. De Oliveira T. (2018). Renewing Felsenstein’s Phylogenetic Bootstrap in the Era of Big Data. Nature 556 (7702), 452–456. 10.1038/s41586-018-0043-0 29670290 PMC6030568

[B130] Letunic I. Bork P. (2016). Interactive Tree of Life (iTOL) V3: an Online Tool for the Display and Annotation of Phylogenetic and Other Trees. Nucleic Acids Res. 44 (W1), W242–W245. 10.1093/nar/gkw290 27095192 PMC4987883

[B131] Letunic I. Bork P. (2019). Interactive Tree of Life (iTOL) V4: Recent Updates and New Developments. Nucleic Acids Res. 47 (W1), W256–W259. 10.1093/nar/gkz239 30931475 PMC6602468

[B132] Li H. Durbin R. (2010). Fast and Accurate Long-Read Alignment with Burrows-Wheeler Transform. Bioinformatics 26 (5), 589–595. 10.1093/bioinformatics/btp698 20080505 PMC2828108

[B133] Li H. Durbin R. (2009). Fast and Accurate Short Read Alignment with Burrows-Wheeler Transform. Bioinformatics 25 (14), 1754–1760. 10.1093/bioinformatics/btp324 19451168 PMC2705234

[B134] Li H. (2015). Microbiome, Metagenomics, and High-Dimensional Compositional Data Analysis. Annu. Rev. Stat. Appl. 2 (1), 73–94. 10.1146/annurev-statistics-010814-020351

[B135] Liede-Schumann S. Grimm G. W. Nürk N. M. Potts A. J. Meve U. Hartmann H. E. K. (2020). Phylogenetic Relationships in the Southern African Genus Drosanthemum (Ruschioideae, Aizoaceae). PeerJ 8 (3), e8999. 10.7717/peerj.8999 32426182 PMC7213013

[B136] Lin H. Peddada S. D. (2020). Analysis of Microbial Compositions: a Review of Normalization and Differential Abundance Analysis. NPJ Biofilms Microbiomes 61 (1), 601–613. 10.1038/s41522-020-00160-w PMC7710733 33268781

[B137] Linard B. Romashchenko N. Pardi F. Rivals E. (2020). PEWO: a Collection of Workflows to Benchmark Phylogenetic Placement. Bioinformatics. 10.1093/bioinformatics/btaa657 32697844

[B138] Linard B. Swenson K. Pardi F. (2019). Rapid Alignment-free Phylogenetic Identification of Metagenomic Sequences. Bioinformatics 35 (18), 3303–3312. 10.1093/bioinformatics/btz068 30698645

[B139] Lindgreen S. Adair K. L. Gardner P. P. (2016). An Evaluation of the Accuracy and Speed of Metagenome Analysis Tools. Sci. Rep. 6 (1), 19233. 10.1038/srep19233 26778510 PMC4726098

[B140] Liu K. Warnow T. J. Holder M. T. Nelesen S. M. Yu J. Stamatakis A. P. (2012). SATe-II: Very Fast and Accurate Simultaneous Estimation of Multiple Sequence Alignments and Phylogenetic Trees. Syst. Biol. 61 (1), 90–106. 10.1093/sysbio/syr095 22139466

[B141] Logares R. Haverkamp T. H. Kumar S. Lanzén A. Nederbragt A. J. Quince C. (2012). Environmental Microbiology through the Lens of High-Throughput DNA Sequencing: Synopsis of Current Platforms and Bioinformatics Approaches. J. Microbiol. Methods 91 (1), 106–113. 10.1016/j.mimet.2012.07.017 22849829

[B142] Logares R. Sunagawa S. Salazar G. Cornejo-Castillo F. M. Ferrera I. Sarmento H. (2014). Metagenomic 16S rDNA Illumina Tags Are a Powerful Alternative to Amplicon Sequencing to Explore Diversity and Structure of Microbial Communities. Environ. Microbiol. 16 (9), 2659–2671. 10.1111/1462-2920.12250 24102695

[B143] López-García A. Pineda-Quiroga C. Atxaerandio R. Adrian P. Hernández I. García-Rodríguez A. (2018). Comparison of Mothur and QIIME for the Analysis of Rumen Microbiota Composition Based on 16S rRNA Amplicon Sequences. Front. Microbiol. 9 (DEC), 1–11. 10.3389/fmicb.2018.03010 30619117 PMC6300507

[B144] Lorimer J. Hodgetts T. Grenyer R. Greenhough B. McLeod C. Dwyer A. (2019). Making the Microbiome Public: Participatory Experiments with DNA Sequencing in Domestic Kitchens. Trans. Inst. Br. Geogr. 44 (3), 524–541. 10.1111/tran.12289

[B145] Love M. I. Hogenesch J. B. Irizarry R. A. (2016). Modeling of RNA-Seq Fragment Sequence Bias Reduces Systematic Errors in Transcript Abundance Estimation. Nat. Biotechnol. 34 (12), 1287–1291. 10.1038/nbt.3682 27669167 PMC5143225

[B146] Löytynoja A. Vilella A. J. Goldman N. (2012). Accurate Extension of Multiple Sequence Alignments Using a Phylogeny-Aware Graph Algorithm. Bioinformatics 28 (13), 1684–1691. 10.1093/bioinformatics/bts198 22531217 PMC3381962

[B147] Lozupone C. Knight R. (2005). UniFrac: a New Phylogenetic Method for Comparing Microbial Communities. Appl. Environ. Microbiol. 71 (12), 8228–8235. 10.1128/AEM.71.12.8228-8235.2005 16332807 PMC1317376

[B148] Lozupone C. A. Hamady M. Kelley S. T. Knight R. (2007). Quantitative and Qualitative Beta Diversity Measures lead to Different Insights into Factors that Structure Microbial Communities. Appl. Environ. Microbiol. 73 (5), 1576–1585. 10.1128/AEM.01996-06 17220268 PMC1828774

[B151] Mahé F. de Vargas C. Bass D. Czech L. Stamatakis A. Lara E. (2017). Parasites Dominate Hyperdiverse Soil Protist Communities in Neotropical Rainforests. Nat. Ecol. Evol. 1 (4), 91. 10.1038/s41559-017-0091 28812652

[B152] Mahé F. Czech L. Stamatakis A. Quince C. de Vargas C. Dunthorn M. (2021). Swarm V3: towards Tera-Scale Amplicon Clustering. Bioinformatics 38 (1), 267–269. 10.1093/bioinformatics/btab493 PMC8696092 34244702

[B153] Mardis E. R. (2016). DNA Sequencing Technologies: 2006-2016. Nat. Protoc. 12 (2), 213–218. 10.1038/nprot.2016.182 28055035

[B154] Mardis E. R. (2013). Next-generation Sequencing Platforms. Annu. Rev. Anal. Chem. (Palo Alto Calif. 6 (1), 287–303. 10.1146/annurev-anchem-062012-092628 23560931

[B155] Martiniano R. De Sanctis B. Hallast P. Durbin R. (2022). Placing Ancient DNA Sequences into Reference Phylogenies. Mol. Biol. Evol. 39 (2), msac017. 10.1093/molbev/msac017 35084493 PMC8857924

[B156] Matsen F. A. Evans S. N. (2013). Edge Principal Components and Squash Clustering: Using the Special Structure of Phylogenetic Placement Data for Sample Comparison. PLOS ONE 8 (3), e56859–17. 10.1371/journal.pone.0056859 23505415 PMC3594297

[B157] Matsen F. A. Gallagher A. (2012). Reconciling Taxonomy and Phylogenetic Inference: Formalism and Algorithms for Describing Discord and Inferring Taxonomic Roots. Algorithms Mol. Biol. 7 (1), 8. 10.1186/1748-7188-7-8 22549005 PMC3384453

[B158] Matsen F. A. Hoffman N. G. Gallagher A. Stamatakis A. (2012). A Format for Phylogenetic Placements. PLoS ONE 7 (2), e31009–4. 10.1371/journal.pone.0031009 22383988 PMC3284489

[B159] Matsen F. A. Kodner R. B. Armbrust E. V. (2010). Pplacer: Linear Time Maximum-Likelihood and Bayesian Phylogenetic Placement of Sequences onto a Fixed Reference Tree. BMC Bioinformatics 11 (1), 538. 10.1186/1471-2105-11-538 21034504 PMC3098090

[B160] Matsen F. A. (2015). Phylogenetics and the Human Microbiome. Syst. Biol. 64 (1). 10.1093/sysbio/syu053 PMC4265140 25102857

[B161] McCoy C. O. Matsen F. A. (2013). Abundance-weighted Phylogenetic Diversity Measures Distinguish Microbial Community States and Are Robust to Sampling Depth. PeerJ 1, e157. 10.7717/peerj.157 24058885 PMC3775626

[B162] McDonald D. Price M. N. Goodrich J. Nawrocki E. P. Desantis T. Z. Probst A. (2012). An Improved Greengenes Taxonomy with Explicit Ranks for Ecological and Evolutionary Analyses of Bacteria and Archaea. ISME J. 6 (3), 610–618. 10.1038/ismej.2011.139 22134646 PMC3280142

[B186] McMurdie P. J. Holmes S. (2014). Waste Not, Want Not: Why Rarefying Microbiome Data Is Inadmissible. PLoS Comput. Biol. 10 (4), e1003531. 10.1371/journal.pcbi.1003531 24699258 PMC3974642

[B163] Menzel P. Ng K. L. Krogh A. (2016). Fast and Sensitive Taxonomic Classification for Metagenomics with Kaiju. Nat. Commun. 7 (1), 11257–11259. 10.1038/ncomms11257 27071849 PMC4833860

[B164] Methé B. A. Nelson K. E. Pop M. Creasy H. H. Giglio M. G. Curtis H. (2012). A Framework for Human Microbiome Research. Nature 486 (7402), 215–221. 10.1038/nature11209 22699610 PMC3377744

[B165] Metin B. Jiang Y. Roush D. Zhu Q. Mirarab S. (2021). Fast and Accurate Distance-Based Phylogenetic Placement Using divide and Conquer. Mol. Ecol. Resour. 22 (3), 1213–1227. 10.1111/1755-0998 34643995

[B166] Metin B. Sarmashghi S. Mirarab S. (2019). APPLES: Scalable Distance-Based Phylogenetic Placement with or without Alignments. Syst. Biol. 10.1093/sysbio/syz063/5572672 PMC7164367 31545363

[B167] Meyer A. Todt C. Mikkelsen N. T. Lieb B. (2010). Fast Evolving 18S rRNA Sequences from Solenogastres (Mollusca) Resist Standard PCR Amplification and Give New Insights into Mollusk Substitution Rate Heterogeneity. BMC Evol. Biol. 110 (1), 70. 10.1186/1471-2148-10-70 PMC2841657 20214780

[B168] Meyer F. Bremges A. Belmann P. Janssen S. McHardy A. C. Koslicki D. (2019). Assessing Taxonomic Metagenome Profilers with OPAL. Genome Biol. 20 (1), 51. 10.1186/s13059-019-1646-y 30832730 PMC6398228

[B169] Mignardi M. Nilsson M. (2014). Fourth-generation Sequencing in the Cell and the Clinic. Genome Med. 6 (4), 31. 10.1186/gm548 25031621 PMC4062057

[B170] Mirarab S. Nguyen N. Warnow T. (2012). “SEPP: SATé-Enabled Phylogenetic Placement,” in Pacific Symposium on Biocomputing (World Scientific), 247–258. 10.1142/9789814366496_0024 22174280

[B171] Morel B. Barbera P. Czech L. Bettisworth B. Hübner L. Lutteropp S. (2020). Phylogenetic Analysis of SARS-CoV-2 Data Is Difficult. Mol. Biol. Evol. 38 (5), 1777–1791. 10.1093/molbev/msaa314 PMC7798910 33316067

[B172] Moret B. M. E. Roshan U. Warnow T. (2002). “Sequence-length Requirements for Phylogenetic Methods,” in Lecture Notes in Computer Science. Editors Guigó R. Gusfield D. (Berlin, Heidelberg: Springer Berlin Heidelberg), 2452, 343–356. 3540442111. 10.1007/3-540-45784-4_26

[B173] Morgan J. L. Darling A. E. Eisen J. A. (2010). Metagenomic Sequencing of an In Vitro-simulated Microbial Community. PLoS ONE 5 (4), e10209–10. 10.1371/journal.pone.0010209 20419134 PMC2855710

[B174] Morgan-Lang C. McLaughlin R. Armstrong Z. Zhang G. Chan K. Hallam S. J. (2020). TreeSAPP: the Tree-Based Sensitive and Accurate Phylogenetic Profiler. Bioinformatics 36 (18), 4706–4713. 10.1093/bioinformatics/btaa588 32637989 PMC7695126

[B175] Mühlemann B. Vinner L. Margaryan A. Wilhelmson H. De La Fuente Castro C. Allentoft M. E. (2020). Diverse variola Virus (Smallpox) Strains Were Widespread in Northern Europe in the Viking Age. Science 369 (6502). 10.1126/science.aaw8977 32703849

[B176] Muir P. Li S. Lou S. Wang D. Spakowicz D. J. Salichos L. (2016). Erratum to: The Real Cost of Sequencing: Scaling Computation to Keep Pace with Data Generation. Genome Biol. 17 (1), 78–79. 10.1186/s13059-016-0961-9 27125642 PMC4850727

[B177] Nguyen L. T. Schmidt H. A. Von Haeseler A. Minh B. Q. (2015). IQ-TREE: a Fast and Effective Stochastic Algorithm for Estimating Maximum-Likelihood Phylogenies. Mol. Biol. Evol. 32 (1), 268–274. 10.1093/molbev/msu300 25371430 PMC4271533

[B178] Nguyen N. P. Mirarab S. Liu B. Pop M. Warnow T. (2014). TIPP: Taxonomic Identification and Phylogenetic Profiling. Bioinformatics 30 (24), 3548–3555. 10.1093/bioinformatics/btu721 25359891 PMC4253836

[B179] Niedringhaus T. P. Milanova D. Kerby M. B. Snyder M. P. Barron A. E. (2011). Landscape of Next-Generation Sequencing Technologies. Anal. Chem. 83 (12), 4327–4341. 10.1021/ac2010857 21612267 PMC3437308

[B180] Notredame C. Higgins D. G. Heringa J. (2000). T-coffee: a Novel Method for Fast and Accurate Multiple Sequence Alignment. J. Mol. Biol. 302 (1), 205–217. 10.1006/jmbi.2000.4042 10964570

[B181] Nugent R. P. Krohn M. A. Hillier S. L. (1991). Reliability of Diagnosing Bacterial Vaginosis Is Improved by a Standardized Method of Gram Stain Interpretation. J. Clin. Microbiol. 29 (2), 297–301. 10.1128/JCM.29.2.297-301.1991 1706728 PMC269757

[B182] Ondov B. D. Bergman N. H. Phillippy A. M. (2011). Interactive Metagenomic Visualization in a Web Browser. BMC Bioinformatics 12 (1), 385. 10.1186/1471-2105-12-385 21961884 PMC3190407

[B183] Oulas A. Pavloudi C. Polymenakou P. Pavlopoulos G. A. Papanikolaou N. Kotoulas G. (2015). Metagenomics: Tools and Insights for Analyzing Next-Generation Sequencing Data Derived from Biodiversity Studies. Bioinform Biol. Insights 9 (75–88), 75–88. 10.4137/BBI.S12462 25983555 PMC4426941

[B184] Pareek C. S. Smoczynski R. Tretyn A. (2011). Sequencing Technologies and Genome Sequencing. J. Appl. Genet. 52 (4), 413–435. 10.1007/s13353-011-0057-x 21698376 PMC3189340

[B185] Parks D. H. Rinke C. Chuvochina M. Chaumeil P. A. Woodcroft B. J. Evans P. N. (2017). Recovery of Nearly 8,000 Metagenome-Assembled Genomes Substantially Expands the Tree of Life. Nat. Microbiol. 2 (11), 1533–1542. 10.1038/s41564-017-0012-7 28894102

[B187] Pawlowsky-Glahn V. Egozcue J. J. Tolosana-Delgado R. (2015). Modeling and Analysis of Compositional Data. Chichester, UK: John Wiley & Sons.

[B188] Peabody M. A. Van Rossum T. Lo R. Brinkman F. S. (2015). Evaluation of Shotgun Metagenomics Sequence Classification Methods Using In Silico and *In Vitro* Simulated Communities. BMC Bioinformatics 16, 363. 10.1186/s12859-015-0788-5 PMC4634789 26537885

[B189] Pearson W. R. Lipman D. J. (1988). Improved Tools for Biological Sequence Comparison. Proc. Natl. Acad. Sci. U S A. 85 (8), 2444–2448. 10.1073/pnas.85.8.2444 3162770 PMC280013

[B48] Pelleg D. Moore A. W. (2000). X-means: Extending K-Means with Efficient Estimation of the Number of Clusters. ICML 1, 727–734.

[B190] Peng X. Li G. Liu Z. (2016). Zero-Inflated Beta Regression for Differential Abundance Analysis with Metagenomics Data. J. Comput. Biol. 23 (2), 102. 10.1089/cmb.2015.0157 26675626 PMC6109378

[B191] Pereira-Flores E. Glöckner F. O. Fernandez-Guerra A. (2019). Fast and Accurate Average Genome Size and 16s rRNA Gene Average Copy Number Computation in Metagenomic Data. BMC Bioinformatics 20 (1), 453. 10.1186/s12859-019-3031-y 31488068 PMC6727555

[B192] Pervez M. T. Babar M. E. Nadeem A. Aslam M. AwanAwan A. R. Aslam N. (2014). Evaluating the Accuracy and Efficiency of Multiple Sequence Alignment Methods. Evol. Bioinform Online 10, 205–217. 10.4137/EBO.S19199 25574120 PMC4267518

[B193] Petrenko P. Lobb B. Kurtz D. A. Neufeld J. D. Doxey A. C. (2015). MetAnnotate: Function-specific Taxonomic Profiling and Comparison of Metagenomes. BMC Biol. 13 (1), 92. 10.1186/s12915-015-0195-4 26541816 PMC4636000

[B194] Pettersson E. Lundeberg J. Ahmadian A. (2009). Generations of Sequencing Technologies. Genomics 93 (2), 105–111. 10.1016/j.ygeno.2008.10.003 18992322

[B195] Piredda R. Grimm G. W. Schulze E. D. Denk T. Simeone M. C. (2021). High-throughput Sequencing of 5S-IGS in oaks: Exploring Intragenomic Variation and Algorithms to Recognize Target Species in Pure and Mixed Samples. Mol. Ecol. Resour. 21 (2), 495–510. 10.1111/1755-0998.13264 32997899

[B196] Price M. N. Dehal P. S. Arkin A. P. (2010). FastTree 2-approximately Maximum-Likelihood Trees for Large Alignments. PLoS ONE 5 (3), e9490. 10.1371/journal.pone.0009490 20224823 PMC2835736

[B197] Prodan A. Tremaroli V. Brolin H. Zwinderman A. H. Nieuwdorp M. Levin E. (2020). Comparing Bioinformatic Pipelines for Microbial 16S rRNA Amplicon Sequencing. PLoS ONE 15 (1), e0227434–19. 10.1371/journal.pone.0227434 31945086 PMC6964864

[B198] Pruesse E. Quast C. Knittel K. Fuchs B. M. Ludwig W. Peplies J. (2007). SILVA: a Comprehensive Online Resource for Quality Checked and Aligned Ribosomal RNA Sequence Data Compatible with ARB. Nucleic Acids Res. 35 (21), 7188–7196. 10.1093/nar/gkm864 17947321 PMC2175337

[B199] Quast C. Pruesse E. Yilmaz P. Gerken J. Schweer T. Yarza P. (2013). The SILVA Ribosomal RNA Gene Database Project: Improved Data Processing and Web-Based Tools. Nucleic Acids Res. 41 (D1), D590–D596. 10.1093/nar/gks1219 23193283 PMC3531112

[B200] Quinn T. P. Erb I. Richardson M. F. Crowley T. M. (2018). Understanding Sequencing Data as Compositions: an Outlook and Review. Bioinformatics 34 (16), 2870–2878. 10.1093/bioinformatics/bty175 29608657 PMC6084572

[B201] Rabiee M. Mirarab S. (2019). INSTRAL: Discordance-Aware Phylogenetic Placement Using Quartet Scores. Syst. Biol. 69 (2), 384–391. 10.1093/sysbio/syz045 31290974

[B202] Rajter Ľ. Dunthorn M. (2021). Ciliate SSU-rDNA Reference Alignments and Trees for Phylogenetic Placements of Metabarcoding Data. Metabarcoding and Metagenomics 5, e69602. 10.3897/mbmg.5.69602

[B203] Rajter Ľ. Ewers I. Graupner N. Vďačný P. Dunthorn M. (2021). Colpodean Ciliate Phylogeny and Reference Alignments for Phylogenetic Placements. Eur. J. Protistol 77, 125747. 10.1016/j.ejop.2020.125747 33279755

[B204] Ren R. Sun Y. Zhao Y. Geiser D. Ma H. Zhou X. (2016). Phylogenetic Resolution of Deep Eukaryotic and Fungal Relationships Using Highly Conserved Low-Copy Nuclear Genes. Genome Biol. Evol. 8 (9), 2683–2701. 10.1093/gbe/evw196 27604879 PMC5631032

[B205] Reuter J. A. Spacek D. V. Snyder M. P. (2015). High-Throughput Sequencing Technologies. Mol. Cel 58 (4), 586–597. 10.1016/j.molcel.2015.05.004 PMC4494749 26000844

[B206] Ritter C. D. Dunthorn M. Anslan S. de Lima V. X. Tedersoo L. Nilsson R. H. (2020). Advancing Biodiversity Assessments with Environmental DNA: Long-Read Technologies Help Reveal the Drivers of Amazonian Fungal Diversity. Ecol. Evol. 10 (14), 7509–7524. 10.1002/ece3.6477 32760545 PMC7391351

[B207] Rognes T. Flouri T. Nichols B. Quince C. Mahé F. (2016). VSEARCH: a Versatile Open Source Tool for Metagenomics. PeerJ 4, e2584. 10.7717/peerj.2584 27781170 PMC5075697

[B208] Ronquist F. (2004). Bayesian Inference of Character Evolution. Trends Ecol. Evol. 19 (9), 475–481. 10.1016/j.tree.2004.07.002 16701310

[B224] Rousseeuw P. J. (1987). Silhouettes: A Graphical Aid to the Interpretation and Validation of Cluster Analysis. J. Comput. Appl. Math. 20, 53–65. 10.1016/0377-0427(87)90125-7

[B209] Rubinat-Ripoll L. (2019). Lrubinat/Photoreft: A 16s Rdna Reference Tree Representing the Main Groups of Picophototrophic Eukaryotes and Prokaryotes. Available at: https://zenodo.org/record/3476953 .

[B210] Ruppert K. M. Kline R. J. Rahman M. S. (2019). Past, Present, and Future Perspectives of Environmental Dna (edna) Metabarcoding: A Systematic Review in Methods, Monitoring, and Applications of Global edna. Glob. Ecol. Conservation 17, e00547. 10.1016/j.gecco.2019.e00547

[B211] Saitou N. Nei M. (1987). The Neighbor-Joining Method: A New Method for Reconstructing Phylogenetic Trees. Mol. Biol. Evol. 4 (4), 406–425. 10.1093/oxfordjournals.molbev.a040454 3447015

[B212] Sankoff D. (1975). Minimal Mutation Trees of Sequences. SIAM J. Appl. Math. 28 (1), 35–42. 10.1137/0128004

[B246] Savolainen V. Cowan R. S. Vogler A. P. Roderick G. K. Lane R. (2005). Towards Writing the Encyclopedia of Life: An Introduction to DNA Barcoding. Philos. Trans. R. Soc. Lond. B Biol. Sci. 360 (1462), 1805–1811. 10.1098/rstb.2005.1730 16214739 PMC1609222

[B213] Sayers E. W. Barrett T. Benson D. A. Bryant S. H. Canese K. Chetvernin V. (2009). Database Resources of the National Center for Biotechnology Information. Nucleic Acids Res. 37, D5–D15. 10.1093/nar/gkn741 18940862 PMC2686545

[B214] Schloss P. D. Westcott S. L. Ryabin T. Hall J. R. Hartmann M. Hollister E. B. (2009). Introducing Mothur: Open-Source, Platform-independent, Community-Supported Software for Describing and Comparing Microbial Communities. Appl. Environ. Microbiol. 75 (23), 7537–7541. 10.1128/AEM.01541-09 19801464 PMC2786419

[B215] Schön M. E. Eme L. Ettema T. J. G. (2019). PhyloMagnet: Fast and Accurate Screening of Short-Read Meta-Omics Data Using Gene-Centric Phylogenetics. Bioinformatics 36 (6), 1718–1724. 10.1093/bioinformatics/btz799 PMC7703773 31647547

[B216] Schreiber F. Gumrich P. Daniel R. Meinicke P. (2010). Treephyler: Fast Taxonomic Profiling of Metagenomes. Bioinformatics 26 (7), 960–961. 10.1093/bioinformatics/btq070 20172941

[B217] Sczyrba A. Hofmann P. Belmann P. Koslicki D. Janssen S. Dröge J. (2017). Critical Assessment of Metagenome Interpretation-A Benchmark of Metagenomics Software. Nat. Methods 14 (11), 1063–1071. 10.1038/nmeth.4458 28967888 PMC5903868

[B219] Segata N. Waldron L. Ballarini A. Narasimhan V. Jousson O. Huttenhower C. (2012). Metagenomic Microbial Community Profiling Using Unique Clade-specific Marker Genes. Nat. Methods 9 (8), 811–814. 10.1038/nmeth.2066 22688413 PMC3443552

[B220] Sempéré G. Pétel A. Abbé M. Lefeuvre P. Roumagnac P. Mahé F. (2021). metaXplor: an Interactive Viral and Microbial Metagenomic Data Manager. GigaScience 10 (2), January. 10.1093/gigascience/giab001 PMC7931823 33527143

[B221] Shah N. Molloy E. K. Pop M. Warnow T. (2021). TIPP2: Metagenomic Taxonomic Profiling Using Phylogenetic Markers. Bioinformatics. 10.1093/bioinformatics/btab023 PMC8317105 33471121

[B222] Shah N. Nute M. G. Warnow T. Pop M. (2019). Misunderstood Parameter of NCBI BLAST Impacts the Correctness of Bioinformatics Workflows. Bioinformatics. 10.1093/bioinformatics/bty833 30247621

[B223] Sharon I. Kertesz M. Hug L. A. Pushkarev D. Blauwkamp T. A. Castelle C. J. (2015). Accurate, Multi-Kb Reads Resolve Complex Populations and Detect Rare Microorganisms. Genome Res. 25 (4), 534–543. 10.1101/gr.183012.114 25665577 PMC4381525

[B225] Silverman J. D. Bloom R. J. Jiang S. Durand H. K. Dallow E. Mukherjee S. (2021). Measuring and Mitigating PCR Bias in Microbiota Datasets. Plos Comput. Biol. 17 (7), e1009113. 10.1371/journal.pcbi.1009113 34228723 PMC8284789

[B226] Silverman J. D. Washburne A. D. Mukherjee S. Lawrence A. D. (2017). A Phylogenetic Transform Enhances Analysis of Compositional Microbiota Data. eLife 6, e21887. 10.7554/eLife.21887 28198697 PMC5328592

[B227] Simão F. A. Waterhouse R. M. Ioannidis P. Kriventseva E. V. Zdobnov E. M. (2015). BUSCO: Assessing Genome Assembly and Annotation Completeness with Single-Copy Orthologs. Bioinformatics 31 (19), 3210–3212. 10.1093/bioinformatics/btv351 26059717

[B228] Smith S. A. Pease J. B. (2017). Heterogeneous Molecular Processes Among the Causes of How Sequence Similarity Scores Can Fail to Recapitulate Phylogeny. Brief Bioinform 18 (3), 451–457. 10.1093/bib/bbw034 27103098 PMC5429007

[B229] Srinivasan S. Hoffman N. G. Morgan M. T. Matsen F. A. Fiedler T. L. Hall R. W. (2012). Bacterial Communities in Women with Bacterial Vaginosis: High Resolution Phylogenetic Analyses Reveal Relationships of Microbiota to Clinical Criteria. PLOS ONE 7 (6), e37818. 10.1371/journal.pone.0037818 22719852 PMC3377712

[B230] Stamatakis A. (2014). RAxML Version 8: a Tool for Phylogenetic Analysis and post-analysis of Large Phylogenies. Bioinformatics 30 (9), 1312–1313. 10.1093/bioinformatics/btu033 24451623 PMC3998144

[B231] Stark M. Berger S. A. Stamatakis A. von Mering C. (2010). MLTreeMap-accurate Maximum Likelihood Placement of Environmental DNA Sequences into Taxonomic and Functional Reference Phylogenies. BMC Genomics 11 (1), 461. 10.1186/1471-2164-11-461 20687950 PMC3091657

[B232] Strimmer K. Rambaut A. (2002). Inferring Confidence Sets of Possibly Misspecified Gene Trees. Proc. Biol. Sci. 269, 137–142. 10.1098/rspb.2001.1862 11798428 PMC1690879

[B233] Sunagawa S. Mende D. R. Zeller G. Izquierdo-Carrasco F. Berger S. A. Kultima J. R. (2013). Metagenomic Species Profiling Using Universal Phylogenetic Marker Genes. Nat. Methods 10 (12), 1196–1199. 10.1038/nmeth.2693 24141494

[B234] Temperton B. Giovannoni S. J. Metagenomics G. (2012). Metagenomics: Microbial Diversity through a Scratched Lens. Curr. Opin. Microbiol. 15 (5), 605–612. 10.1016/j.mib.2012.07.001 22831844

[B236] Thomas T. Gilbert J. Meyer F. (2012). Metagenomics - a Guide from Sampling to Data Analysis. Microb. Inform. Exp. 2 (1), 3. 10.1186/2042-5783-2-3 22587947 PMC3351745

[B237] Thorndike R. L. (1953). Who Belongs in the Family? Psychometrika 18 (4), 267–276. 10.1007/bf02289263

[B238] Tibshirani R. Walther G. Hastie T. (2001). Estimating the Number of Clusters in a Data Set via the gap Statistic. J. R. Stat. Soc. Ser. B (Statistical Methodology) 63 (2), 411–423. 10.1111/1467-9868.00293

[B240] Truong D. T. Franzosa E. A. Tickle T. L. Scholz M. Weingart G. Pasolli E. (2015). MetaPhlAn2 for Enhanced Metagenomic Taxonomic Profiling. Nat. Methods 12 (10), 902–903. 10.1038/nmeth.3589 26418763

[B241] Tsilimigras M. C. B. Fodor A. A. (2016). Compositional Data Analysis of the Microbiome: Fundamentals, Tools, and Challenges. Ann. Epidemiol. 26 (5), 330–335. 10.1016/j.annepidem.2016.03.002 27255738

[B242] Tucker C. M. Cadotte M. W. Carvalho S. B. Davies T. J. Ferrier S. Fritz S. A. (2017). A Guide to Phylogenetic Metrics for Conservation, Community Ecology and Macroecology. Biol. Rev. Camb Philos. Soc. 92 (2), 698–715. 10.1111/brv.12252 26785932 PMC5096690

[B243] Turakhia Y. Thornlow B. Hinrichs A. S. De Maio N. Gozashti L. Lanfear R. (2021). Ultrafast Sample Placement on Existing tRees (UShER) Enables Real-Time Phylogenetics for the SARS-CoV-2 Pandemic. Nat. Genet. 53 (6), 809–816. 10.1038/s41588-021-00862-7 33972780 PMC9248294

[B244] Tyson G. W. Chapman J. Hugenholtz P. Allen E. E. Ram R. J. Richardson P. M. (2020). Community Structure and Metabolism through Reconstruction of Microbial Genomes from the Environment. Nature 428, 37–43. 10.1038/nature02340 14961025

[B245] van Dijk E. L. Auger H. Jaszczyszyn Y. Thermes C. (2014). Ten Years of Next-Generation Sequencing Technology. Trends Genet. 30 (9), 418–426. 10.1016/j.tig.2014.07.001 25108476

[B247] von Mering C. Hugenholtz P. Raes J. Tringe S. G. Doerks T. Jensen L. J. (2007). Quantitative Phylogenetic Assessment of Microbial Communities in Diverse Environments. Science 315 (5815), 1126–1130. 10.1126/science.1133420 17272687

[B248] Wang L. G. Lam T. T. Xu S. Dai Z. Zhou L. Feng T. (2020). Treeio: An R Package for Phylogenetic Tree Input and Output with Richly Annotated and Associated Data. Mol. Biol. Evol. 37 (2), 599–603. 10.1093/molbev/msz240 31633786 PMC6993851

[B249] Wang Q. Garrity G. M. Tiedje J. M. Cole J. R. (2007). Naive Bayesian Classifier for Rapid Assignment of rRNA Sequences into the New Bacterial Taxonomy. Appl. Environ. Microbiol. 73 (16), 5261–5267. 10.1128/AEM.00062-07 17586664 PMC1950982

[B250] Wang W. L. Xu S. Y. Ren Z. G. Tao L. Jiang J. W. Zheng S. S. (2015). Application of Metagenomics in the Human Gut Microbiome. World J. Gastroenterol. 21 (3), 803–814. 10.3748/wjg.v21.i3.803 25624713 PMC4299332

[B251] Washburne A. D. Silverman J. D. Leff J. W. Dominic J. Bennett J. L. D. Mukherjee S. (2017). Phylogenetic Factorization of Compositional Data Yields Lineage-Level Associations in Microbiome Datasets. PeerJ 5, e2969. 10.7717/peerj.2969 28289558 PMC5345826

[B252] Washburne A. D. Silverman J. D. Morton J. T. Becker D. J. Crowley D. Mukherjee S. (2019). Phylofactorization: a Graph Partitioning Algorithm to Identify Phylogenetic Scales of Ecological Data. Ecol. Monogr. 89 (2), e01353. 10.1002/ecm.1353

[B253] Wedell E. Cai Y. Warnow T. (2021). “Scalable and Accurate Phylogenetic Placement Using Pplacer-XR,” in International Conference on Algorithms for Computational Biology (Springer), 94–105. 10.1007/978-3-030-74432-8_7

[B254] Weisburg W. G. Barns S. M. Pelletier D. A. Lane D. J. (1991). 16S Ribosomal DNA Amplification for Phylogenetic Study. J. Bacteriol. 173 (2), 697–703. 10.1128/jb.173.2.697-703.1991 1987160 PMC207061

[B255] Weiss S. Xu Z. Z. Peddada S. Amir A. Bittinger K. Gonzalez A. (2017). Normalization and Microbial Differential Abundance Strategies Depend upon Data Characteristics. Microbiome 5 (1), 27. 10.1186/s40168-017-0237-y 28253908 PMC5335496

[B256] Westcott S. L. Schloss P. D. (2015). De Novo clustering Methods Outperform Reference-Based Methods for Assigning 16S rRNA Gene Sequences to Operational Taxonomic Units. PeerJ 3 (12), e1487. 10.7717/peerj.1487 26664811 PMC4675110

[B257] Woese C. R. Fox G. E. (1977). Phylogenetic Structure of the Prokaryotic Domain: the Primary Kingdoms. Proc. Natl. Acad. Sci. U S A. 74 (11), 5088–5090. 10.1073/pnas.74.11.5088 270744 PMC432104

[B258] Woese C. R. Kandler O. Wheelis M. L. (1990). Towards a Natural System of Organisms: Proposal for the Domains Archaea, Bacteria, and Eucarya. Proc. Natl. Acad. Sci. U S A. 87 (12), 4576–4579. 10.1073/pnas.87.12.4576 2112744 PMC54159

[B259] Wood D. E. Lu J. Langmead B. (2019). Improved Metagenomic Analysis with Kraken 2. Genome Biol. 20 (1), 1–13. 10.1186/s13059-019-1891-0 31779668 PMC6883579

[B260] Wood D. E. Salzberg S. L. Heidelberg J. Halpern A. Rusch D. Eisen J. (2014). Kraken: Ultrafast Metagenomic Sequence Classification Using Exact Alignments. Genome Biol. 15 (3), R46. 10.1186/gb-2014-15-3-r46 24580807 PMC4053813

[B261] Wu M. Scott A. J. (2012). Phylogenomic Analysis of Bacterial and Archaeal Sequences with AMPHORA2. Bioinformatics 28 (7), 1033–1034. 10.1093/bioinformatics/bts079 22332237

[B262] Yang Z. (2006). Computational Molecular Evolution. Oxford University Press.

[B263] Ye S. H. Siddle K. J. Park D. J. Sabeti P. C. (2019). Benchmarking Metagenomics Tools for Taxonomic Classification. Cell 178 (4), 779–794. 10.1016/j.cell.2019.07.010 31398336 PMC6716367

[B264] Yilmaz P. Parfrey L. W. Yarza P. Gerken J. Pruesse E. Quast C. (2014). The SILVA and "All-Species Living Tree Project (LTP)" Taxonomic Frameworks. Nucleic Acids Res. 42 (D1), D643–D648. 10.1093/nar/gkt1209 24293649 PMC3965112

[B265] Yu G. Smith D. K. Zhu H. Guan Y. Lam T. T. Y. (2017). Ggtree : an R Package for Visualization and Annotation of Phylogenetic Trees with Their Covariates and Other Associated Data. Methods Ecol. Evol. 8 (1), 28–36. 10.1111/2041-210X.12628

[B266] Zhang J. Kapli P. Pavlidis P. Stamatakis A. (2013). A General Species Delimitation Method with Applications to Phylogenetic Placements. Bioinformatics 29 (22), 2869–2876. 10.1093/bioinformatics/btt499 23990417 PMC3810850

[B267] Zheng Q. Bartow-McKenney C. Meisel J. S. Grice E. A. (2018). HmmUFOtu: An HMM and Phylogenetic Placement Based Ultra-fast Taxonomic Assignment and OTU Picking Tool for Microbiome Amplicon Sequencing Studies. Genome Biol. 19 (1), 82. 10.1186/s13059-018-1450-0 29950165 PMC6020470

[B268] Zhou X. Shen X. X. Hittinger C. T. Rokas A. (2018). Evaluating Fast Maximum Likelihood-Based Phylogenetic Programs Using Empirical Phylogenomic Data Sets. Mol. Biol. Evol. 35 (2), 486–503. 10.1093/molbev/msx302 29177474 PMC5850867

[B269] Zou Q. Lin G. Jiang X. Liu X. Zeng X. (2020). Sequence Clustering in Bioinformatics: an Empirical Study. Brief. Bioinform. 21 (1), 1–10. 10.1093/bib/bby090 30239587

